# A Systematic Review of “Helicopter Parenting” and Its Relationship With Anxiety and Depression

**DOI:** 10.3389/fpsyg.2022.872981

**Published:** 2022-05-25

**Authors:** Julia Schønning Vigdal, Kolbjørn Kallesten Brønnick

**Affiliations:** ^1^Department of Welfare and Participation, Western Norway University of Applied Sciences, Sogndal, Norway; ^2^Department of Public Health, Faculty of Health Sciences, University of Stavanger, Stavanger, Norway; ^3^SESAM, Department of Psychiatry, Stavanger University Hospital, Stavanger, Norway

**Keywords:** systematic review, helicopter parenting, overprotective parenting, controlling parenting, parenting style, anxiety, depression

## Abstract

**Background:**

Emerging evidence suggests that overprotective and controlling parenting, often referred to as “helicopter parenting” may have negative implications on the child's mental health such as anxiety and depression. However, no systematic review on the topic exists.

**Objective:**

Conducting a systematic review to identify all studies where the relationship between helicopter parenting and symptoms of anxiety and/or depression have been investigated.

**Method:**

A systematic literature search conducted the 3rd of November 2021 yielded 38 eligible studies. Since helicopter parenting is a fairly new construct, this review considered parental control and overprotective parenting to be dimensions of helicopter parenting and thus, eligible for the study. Study quality was assessed in accordance with Campbells Validity Typology.

**Results:**

The majority of the studies included in this review found a direct relationship between helicopter parenting and symptoms of anxiety and depression. However, validity problems undermine these findings with regarding to assessing the causal relationship between helicopter parenting and these symptoms. There were no longitudinal studies of sufficient quality to determine if helicopter parenting precedes the outcome of anxiety and depression.

**Conclusion:**

Even though the majority of the studies included in this systematic review found a relationship between helicopter parenting and anxiety and depression, the evidence for this relationship is insufficient and must be investigated further. Findings suggest that it is important to include both maternal and paternal parenting style in future studies as they could affect the outcome of anxiety and depression differently.

**Systematic Review Registration:**

PROSPERO 2020 CRD42020167465, https://www.crd.york.ac.uk/prospero/display_record.php?RecordID=167465.

## Introduction

A parenting style characterized by overprotection and control is often referred to as “helicopter parenting” (LeMoyne and Buchanan, 2011; Padilla-Walker and Nelson, 2012; Schiffrin et al., 2014). It has been argued that such overprotective parenting started rising around 1985 due to several factors, amongst them the creation of the parent-supervised and scheduled play-date (Lythcott-Haims, 2015). A study using a nationally representative sample of more than 3600 Americans suggested that child-centered, time-intensive parenting has now become a cultural norm and is pervasive, even across different social classes (Ishizuka, 2019). It has also been argued that such intensive parenting is prevalent on a global scale (Anderson, 2019) and has harmful effects such as an increase in depression (Schiffrin et al., 2014) and anxiety (Spokas and Heimberg, 2009). Thus, helicopter parenting is an important modifiable risk factor with regard to mental health.

A parenting style can be conceptualized as a collection of a parents' attitudes and behaviors communicated toward their children. These attitudes and behaviors create a certain type of emotional climate for the children to live in. In sum then, a parenting style is the expression of strategies that a parent employ in their child rearing (Darling and Steinberg, 1993). This review defines helicopter parenting as a parenting style in which the parent expresses behaviors of overprotectiveness toward the child in a controlling manner. Thereby, also communicating the attitude that the child is lacking in self-care competence and thus needs to be overly protected. The term “helicopter parenting” was first coined in 1990 (Cline and Fay, 2020) to illustrate how parents metaphorically may hover over their children, like helicopters, ready to sweep in and rescue their children from disappointments and painful experiences (Cline and Fay, 2020). Arguably, by doing so they are sending the message to their children that they are incapable of overcoming their own struggles and in need of constant protection from the dangers of the world.

Researchers have consistently argued for the importance of parenting on child development and mental health into adulthood (Belsky, 1984; Gutman and Feinstein, 2010; Waller et al., 2013; Masud et al., 2015). Emerging evidence suggests that helicopter parenting may possibly lead to increased anxiety (Spokas and Heimberg, 2009; Segrin et al., 2013), depression (Schiffrin et al., 2014), prescribed medication and increased recreational consumption of pain pills (LeMoyne and Buchanan, 2011). In addition, helicopter parenting has been linked to a decrease in wellbeing (Schiffrin et al., 2014), self-regulation (Perry et al., 2018), self-efficacy (Reed et al., 2016) and poorer academic outcomes (Schiffrin and Liss, 2017; Luebbe et al., 2018).

The consequences may be dire for the individual themselves, and also when examined on a societal level, helicopter parenting can have serious consequences (Nutt, 2018). Data from the National Survey of Children's Health (ages 6–17 years old) show a 20% increase in diagnosed anxiety between 2007 and 2012 (Nutt, 2018). Twenge et al. (2019) conducted a longitudinal study with a nationally representative sample of US adolescents to investigate trends in mood disorders since the mid-2000. They found a 63% increase in depression from 2009 to 2017 and a 71% increase in reported psychological distress from 2008 to 2017 (ages 18–25). These figures suggest an usually large increase of anxiety and depression in a short period of time. The same trend also applies to students in higher education with the emergency calls to counseling in universities doubling over a 5 year period (Gray, 2015). Thus, this rapid rise in rates of anxiety and depression may mark a major generational change (Haidt and Lukianoff, 2018).

Humans need to encounter physical and mental challenges in order to develop resilience (Haidt and Lukianoff, 2018). Seery et al. (2010) found that that individuals whom had experienced a moderate amount of adversity had better mental health as opposed to those whom had no such experience, or too much experience with hardship. Additionally, those with a moderate experience of adversity not only reported having better mental health, but they were also less likely to be affected by a recent adverse event (Seery et al., 2010). The restriction of exposure to challenges by intervening before a child has struggled or failed undermines the child's effort to establish healthy regulatory strategies (Perry et al., 2018). Consequently, there are fewer regulatory strategies to be called upon when the child is without caregiver assistance (Perry et al., 2018). Thus, it would seem that helicopter parenting could be related to the outcome of anxiety and depression as the goal for helicopter parents is to ensure a life without struggles and suffering for their children (Cline and Fay, 2020). This perfect and pain-free life that helicopter parents strive for is counterproductive since it robs children from experiencing competence and autonomy (Haidt and Lukianoff, 2018; Cline and Fay, 2020).

Self-determination theory supports the idea that helicopter parenting can be harmful. Ryan and Deci (2000) outline three basic needs that are innate in us all. If satisfied, these needs promotes health and wellbeing, but a violation of these needs contributes to pathology and ill-being. The first of these needs is the need for autonomy; feeling like one is free to make one's own choices. Secondly, there is the need for competence, feeling confident in one's abilities and accomplishments. Lastly, is the need of relatedness; feeling that one is part of a genuinely caring relationships (Ryan and Deci, 2000). The self-determination theory argues that an individual cannot thrive without all of these being fulfilled, to the same degree that one cannot thrive with water and not food (Ryan and Deci, 2000). Helicopter parents who are over-controlling could reduce the sense of autonomy and competence in the child, which could again undermine their relationship with their child. Thus, helicopter parenting might violate all the three basic needs of the child.

Different phases of childhood, from infancy to adolescence, require specific levels of protection and control to promote optimal developmental outcomes (Santrock, 2019). Children need a certain level of parental control to learn how to be an effective member of society, but they also need autonomy to develop competence and self-sufficiency (Barber et al., 1994). Parents must continuingly navigate between these polarities to ensure that the most appropriate style of parenting is being applied. Adolescence on the other hand is a period characterized by an increased development of independence (Szwedo et al., 2017). Thus, the tension between how much autonomy to give and how much control to assert becomes much more apparent. Additionally, the transition to adulthood has changed drastically in just a few decades where many young adults now find themselves still living at home whilst unattached romantically (Schneider et al., 2016). Thereby, potentially continuing the violation of the crucial needs of autonomy, relatedness, and competence.

By violating these needs helicopter parents could foster anxiety and depression. Self-efficacy refers to an individual's belief in their ability to persevere in face of difficulties (Bandura, 2010). A low sense of efficacy to exercise control over that which one values can give rise to feelings of futility in several ways, for instance through unfulfilled aspirations (Bandura, 2010). Depression is most likely to arise when personal standards of merit are set well above one's perceived efficacy to attain them (Kanfer and Zeiss, 1983; Bandura et al., 1999). Another factor contributing to depression is the exercise of control over the depressing thoughts themselves (Bandura et al., 1999). Couple this with the tendency helicopter parents have to set unobtainable standards for their children in the endeavor to create an image of perfection for the outside world, and it could be argued that parents reinforce this sense of inadequacy (Cline and Fay, 2020). Therefore, when helicopter parenting is combined with a child's feeling of inefficacy, their chance of experiencing depression increases. Likewise, there is a growing body of empirical support for the theory that lowered child perceived competence is related to higher levels of anxiety (Muris et al., 2003; Teachman and Allen, 2007). If an individual ceases to believe that they can contribute adequately by their actions to reach a desired outcome they will, arguably, have little reason to persevere in the face of the challenges they meet (Bandura, 2010). Thus, a low sense of self-efficacy could also increase anxiety, with challenges perceived as a potential threats. If an individual has a low sense of self-efficacy in addition to parents who reinforce this sense of danger by overprotectiveness, it would only be logical to assume that this could increase anxiety levels even more.

Given the context of increasing levels of anxiety and depression on a societal scale, we need more knowledge about the specific role of helicopter parenting in promoting increasing levels of anxiety and depression. One unresolved issue concerns direction of causality: does helicopter parenting cause maladjustment, or is it rather a parental reaction to child maladjustment in the context of a changed society which may have imposed increased demands on developing children and adolescents? Despite the attention this parenting style is receiving internationally, no systematic review has been conducted on the relationship between helicopter parenting and anxiety and depression. The aim of this study is therefore to identify and assess all studies after 1985 where the relationship between helicopter parenting and anxiety or depression have been investigated. To do so sufficiently the research question has been designed in lines with the PICO(S) framework, an acronym standing for patient/population, intervention, comparison, outcome, and study design (Santos et al., 2007). This study is thereby covering a crucial research gap in the literature. Additionally, this study aims to investigate the validity of the included studies, especially with regard to causation. Cross-sectional studies cannot establish the direction of effect. To establish this relationship one must examine whether the cause preceded the effect and if they are related, i.e., they covary (Shadish et al., 2002). It is therefore of interest to see how well we understand the potential relationship between helicopter parenting and symptoms of anxiety and depression.

## Methods

This systematic review follows the guidelines of PRISMA: Preferred Reporting Items for Systematic Review and Meta-Analysis (Moher et al., 2010).

### Protocol and Registration

Methods of the analysis and inclusion criteria were specified in advanced and documented in a protocol. We modified the protocol during the review regarding inclusion-criteria, as we found that including a pre-adolescent population led to heterogeneity that detracted from the coherence of the review.

### Eligibility Criteria

The eligibility criteria was developed in accordance to the PICO(S) framework, see Table 1 (Santos et al., 2007). For the updated search a few adjustments were made to the eligibility criteria's, the original eligibility criterias can be found in the protocol registered in PROSPERO. Quantitative studies which investigated the relationship between helicopter parenting and anxiety and/or depression were included in the review. This review defines helicopter parenting as a parenting style in which the parent communicates an attitude of overprotectiveness toward the child in a controlling manner. As helicopter parenting is a fairly new term (Padilla-Walker and Nelson, 2012) studies which investigated the overprotective or controlling dimension of helicopter parenting were also included. Since this review is focused on the outcome of anxiety and depression, to be included in the review the studies would have to have a valid and separate measurement of one or both of these outcomes. Studies in which helicopter parenting was a moderator rather than a predictor of the outcome of anxiety or depression were not included. Studies with a restricted, selective sample focusing on a somatic disease, were not eligible to be considered in this review. Adolescence is a period characterized by development of independence (Szwedo et al., 2017) and in adolescence depression and anxiety starts to be expressed in the same way as in adults (Keijsers and Poulin, 2013). Therefore, this review focused on participants whom were in adolescence or older as including pre-adolescent children would introduce heterogenity regarding antecedents and consequential outcomes. Studies in which the participants were younger than 10 years old were not included as adolescence begins at year 10 (World Health Organization, 2021). Additionally, studies employing an experimental design were excluded as this review is concerned with the actual parenting and the anxious or depressive behavior it may cause. Experimentally manipulating parenting behavior in this instance would reduce ecological validity if this lead to the parent suddenly acting in an uncommon and unexpected manner. Studies where parental psychopathology was an area of focus were not included. Studies with the aim to validate questionnaires concerning helicopter parenting will also be included in the review. Due to the fact that it has been argued that a major shift in parental involvement happened in 1985, studies conducted before then were not considered (Lythcott-Haims, 2015). Only articles written in English were included.

**Table 1:** Eligibility criteria in accordance to PICO(S).

**Population**	**Intervention**	**Comparison**	**Outcome**	**Study design**
Children of helicopter parents	Parenting	Not applicable	Anxiety and/or depression	Quantitative, non-experimental studies

### Information Sources

An explorative literature search to discover the best search terms was conducted the 5th of August 2019 in the electronical databases of PubMed and PsychINFO. The actual search used in this review was conducted the 8th of August 2019 in the same electronical databases. The search was updated the 3rd of November 2021.

### Search

The electronic database PsychINFO and PubMed were searched using the search terms (“overprotective parent^*^” OR “helicopter parent^*^” OR “controlling parent^*^”) AND (parenting^*^ OR “parenting style^*^”) AND (anxiety OR depression OR “clinical diagnosis^*^”). The limit of publication year from 1985 to the 8th of August 2019 was added. Additional sources were searched for in the reference lists of the selected studies between September and October 2019. The search was updated the 3rd of November 2021 both in PsychINFO, PubMED as well as potential other references.

### Study Selection

The studies detected in the literature search were imported into EndNote where the screening process ensued. First off, any duplicates where removed. Following this, the titles and abstracts of the studies were scoured through and applied to the eligibility criteria. Studies which were deemed ineligible were removed. This was done by one reviewer, however if that reviewer felt uncertain another reviewer was contacted to discuss whether that particular study was eligible or not. After screening the studies by their titles and abstracts the first reviewer read through all the studies in full text to further confirm eligibility. Following this the first reviewer looked for additional sources by scouring the reference list of the full text studies, including those that met the eligibility criteria and that where not already included. To increase the strength of the review the two reviewers met to go through each full text study collectively. Those studies that which were deemed ineligible in this meeting were excluded and listed with reason for their exclusion.

### Data Extraction

One review author extracted the data into a chart in Excel specifically designed for this study.

### Data Items

Information extracted from each included study on: (1) author, (2) year, (3) title of study, (4) country of origin, (5) purpose of the study, (6) sample, (7) recruitment, (8) recruitment, (9) sample size, (10) age group, (11) study design, (12) measurement, (13) main findings related to helicopter parenting and anxiety/depression, (14) conclusion.

### Validity of Individual Studies

One reviewer assessed the validity of the eligible studies according to Campbell's Validity Typology which considers four different types of validity; internal validity, external validity, statistical conclusion validity and construct validity (Shadish et al., 2002). After the first reviewer had determined the quality of the studies the assessments were discussed with the second reviewer so that consensus regarding the validity could be reached.

### Planned Analyses

Since this review is synthesizing primary studies with the objective to understand the relationship between helicopter parenting and anxiety and depression the synthesis will be that of a narrative nature. The validity of the studies included will also be synthesized in a narrative description.

## Results

**Figure 1:**
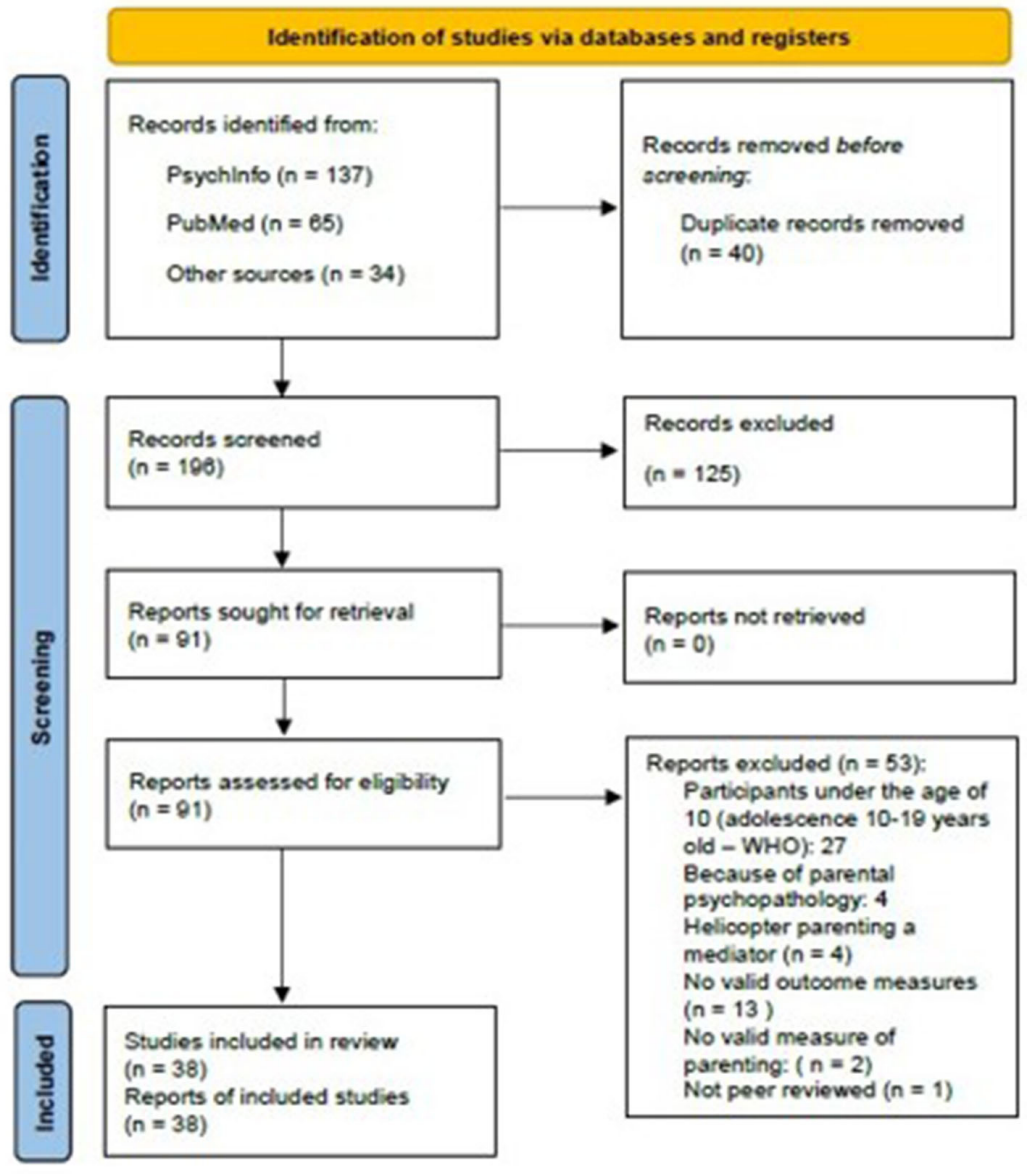
Prisma flow diagram.

**Study selection:** As is illustrated in the PRISMA flow diagram (see Figure 1), 202 studies were identified through the electronic searches in the databases of *PsychINFO* and *Pubmed*. In addition, 34 studies were identified for possible inclusion from hand searching through possible studies' references. Duplicates were removed before any further screening procedure, leaving 196 abstracts and titles to be screened according to the eligibility criteria. This resulted in 91 studies identified for full-text retrieval. After the application of the eligibility criteria to the full-text studies a total amount of 38 studies were selected to be part of the review. Exclusion after full-text retrieval were due to participants being under the age of 10, parental psychopathology, helicopter parenting being a mediator, no valid measure of anxiety or depression, the sample was ineligible or the study was not peer reviewed (see Figure 1).

**Study characteristics:** See Table 2 for a full overview of the study characteristics. The included studies employed a range of different measurements for parenting style and anxiety and depression. In total, 33 cross-sectional studies and 5 longitudinal studies were identified (see Table 2). All 38 studies employed subjective measurements, not researcher observations, for the measurement of helicopter parenting. Thirty-one studies used only child reports and 5 studies used both parent report and child report. The remaining 2 studies employed an additional reporter to the child or parent. A full overview of the different measures used can be found in Table 3.

**Table 2:** Study characteristics.

**Author(s) and year, country**	**Study design**	**Sample description**	**Child age (mean)**	**Measurement**
Basili et al. (2021), Italy, Colombia, and United States	Longitudinal	376 families	12–16 (13.70)	Depression and Anxiety: YSR (CR) Parenting: PPC (PR)
Cai and Tu (2020), United States	Longitudinal	100 adolescent boys and their mothers at time 1	Range not reported (11.05)	Depression: CDI (CR) Anxiety: SASA (CR) Parenting: PBI* (PR)
Cho et al. (2020), Korea	Cross-sectional	233 adolescents and their mothers	13–15 (not reported)	Depression: CES-D (CR) Parenting: MPSS (PR and CR)
Cui et al. (2014), United States	Cross-sectional	206 families with adolescent from disadvantaged communities	10–18 (13.37)	Depression: MFQ-C (CR) Parenting: PCS-YSR (CR)
Cui et al. (2019), United States and Finland	Cross-sectional	747 university students from US and Finland	US (20.45) Finland (22.86)	Depression: CES-D (CR) Anxiety: BAI (CR) Parenting: HPS (CR)
Darlow et al. (2017), United States	Cross-sectional	294 students	18–26 (not reported)	Depression and Anxiety: IPIP (CR) Parenting: PCS (CR), HPS (CR)
Finkelstein et al. (2001), United States	Cross-sectional	111 adolescent girls seeking outpatient mental health services	12–18 (15.65)	Depression: YSR (CR) Parenting: CRPBI (CR)
Finkenauer et al. (2005), Netherlands	Cross-sectional	1,359 adolescents	10–14 (12.3)	Depression: YSR (CR), KDS (CR) Parenting: PSI (CR)
Gargurevich and Soenens (2016), Peru	Cross-sectional	292 late adolescents	16–25 (18.67)	Depression: DEAQ-A (CR), CES-D (CR) Parenting: CRPBI (CR), DAPCS (CR)
Goger et al. (2020), Unites States	Cross-sectional	125 university students	18–25 (18.76)	Anxiety: STAI-T (CR) Parenting: CRPBI (CR)
Hong and Cui (2019)	Cross-sectional	432 college students	18–29 (20.21)	Depression: CES-D (CR) Anxiety: BAI (CR) Parenting: OPS (CR)
Heider et al. (2008), Belgium, France, Germany, Italy, Netherlands, and Spain	Cross-sectional	8,813 adults recruited from the ESEMeD study	18–65+ (47.3)	Anxiety: WMH-CIDI (CR) Parenting: PBI (CR)
Inguglia et al. (2016), Italy and United States	Cross-sectional	908 college students	18–28 Italy (22.11) USA (21.05)	Depression: CES-D (CR) Anxiety: STAI (CR) Parenting: DAPCS (CR)
Klein et al. (2020), Germany	Cross-sectional	8,175 participants from a population based study	Range not reported (59.8)	Depression: PHQ (CR) Anxiety: GAD (CR) Parenting: FEE (CR)
Knappe et al. (2012), Germany^a^	Longitudinal	1,053 adolescents and their parents	14–24 at baseline (not reported)	Anxiety: M-CIDI (CR) Parenting: FEE (CR)
Kouros et al. (2017), United States	Cross-sectional	118 college students	18-25 (19.82)	Anxiety: IDAS (CR) Parenting: HPBQ (CR)
Kullberg et al. (2021), Netherlands^b^	Cross-sectional	636 participants from 256 families. Of each family at least one person with lifetime anxiety and/or depression diagnosis and one sibling. Sample from an ongoing longitudinal cohort study	20-78 (49.7)	Depression: IDS-SR (CR) Anxiety: BAI (CR) Parenting: PBI (CR)
Kullberg et al. (2020), Netherlands^b^	Cross-sectional	2,069 adults with a lifetime affective disorder and healthy controls	26–75 (50.84)	Depression: CIDI (CR), IDS (CR) Anxiety: CIDI (CR), BAI (CR) Parenting: PBI (CR)
LeMoyne and Buchanan (2011), United States	Cross-sectional	317 college students	Range not reported (19.1)	Parenting: HPS (CR)
Levitt et al. (2020), United States	Cross-sectional	117 5th and 6th grade students	Range not reported (11.07)	Depression: CDI (CR) Anxiety: RCMAS (CR) Parenting: P-PASS (CR), PCQ (CR), CRPBI (CR)
Lieb et al. (2000), Germany^a^	Cross-sectional	1,228 adolescents and one of their parents	14–17 (19.7)	Anxiety: M-CIDI (CR) Parenting: FEE (CR)
Luebbe et al. (2018), United States	Cross-sectional	337 university students	17–25 (18.85)	Anxiety and Depression: DASS (CR) Parenting: HP (CR), PBI (CR), PCS-YRS (CR)
Luis et al. (2008), United States and Mexico	Cross-sectional	282 children and their parents	10–14 (not reported)	Anxiety: RCMAS (CR) Parenting: Family discussion task (RR)
Mandara and Pikes (2008), United States	Cross-sectional	152 African American adolescents with lower socioeconomic status	14–18 (17.5)	Depression: CES-D (CR) Parenting: CRPBI (CR), BFPPQ (CR)
Moilanen and Lynn Manuel (2019), United States	Cross-sectional	302 young adults	18–24 (21.57)	Depression: CES-D (CR) Parenting: HPQ (CR), CRPBI (CR)
Overbeek et al. (2007), Netherlands	Longitudinal	4,796 adults whom had participated in a three wave large-scale epidemiological study	18–64 (41.2)	Anxiety and Depression: CIDI (CR) Parenting: PBI (CR)
Reed et al. (2016), United States	Cross-sectional	461 university students	18–25 (19.66)	Anxiety: BAI (CR) Depression: CES-D (CR) Parenting: HPBM (CR)
Reilly and Semkovska (2018), Ireland	Cross-sectional	208 university students	Range not reported (23.36)	Depression: BDI (CR) Parenting: HPS (CR), OPS (CR), HPBQ (CR)
Reitman and Asseff (2010), United States	Cross-sectional	200 college students and their parents	18–35 (19)	Anxiety: STAI (CR, PR) Parenting: CRPBI (CR), PBI (CR)
Rogers et al. (2020), United States	Longitudinal	500 families, including the target adolescent and their parent	10–14 (11.83 at baseline)	Depression: CES-D (CR) Anxiety: SCAI (CR) Parenting: PC-YRS (CR)
Schiffrin et al. (2019), United States	Cross-sectional	446 college students	18–25 (19.59)	Anxiety and Depression: HADS (CR), CES-D (CR) Parenting: CHPS (CR)
Schiffrin et al. (2014), United States	Cross-sectional	297 college students	18–23 (19.34)	Anxiety and Depression: CES-D (CR), HADS (CR) Parenting: OHPQ (CR)
Segrin et al. (2013), United States	Cross-sectional	653 parent-adult child dyads	Range not reported (20.03)	Anxiety: HADS (CR, PR) Parenting: OPS (PR), HPS (CR)
Silove et al. (1991), Australia	Cross-sectional	190 participants where 80 where patients consecutively referred to a hospital out-patient anxiety management programme and 80 were matched controls	19–62 (36.3)	Anxiety: Diagnostic interview including DSM-III-R (RR) Parenting: PBI (CR)
Soenens et al. (2012), Belgia and South-Korea	Cross-sectional	611 high school students	14–18 (16.1)	Depression: CES-D (CR) Parenting: DAPCS, (CR), PCS-YSR (CR), CRPBI (CR)
Turner et al. (2020), United States	Cross-sectional	286 college students	Under the age of 26 (19.2)	Depression: DASS (CR) Parenting: HPS (CR)
Wenze et al. (2019), United States	Cross-sectional	104 undergraduate students	Range not reported (19.15)	Depression: CED-D (CR) Anxiety: GAD (CR) Parenting: HPBQ (CR)
Wu et al. (2018), China	Cross-sectional	373 high school students	13–15 (13.79)	Anxiety: PPK-TC: GELOPH-TC (CR) Parenting: PBI (CR)

*Key: CR, children report; PR, parent report; RR, researcher report*.

a *Study utilized the same sample*.

b *Study utilized the same sample*.

**Table 3:** Overview of measurements used in the included studies.

**Measurement**	**Key**	**References**
BAI	Beck Anxiety Inventory	Beck et al., 1988
BDI	The Beck Depression Inventory	Beck et al., 1996
CES-D	Center for Epidemiologic Studies Depression Scale	Radloff, 1977
CDI	Childhood Depression Inventory	Kovacs and Preiss, 1992
CIDI	Composite International Diagnostic Interview	Smeets and Dingemans, 1993
CRPBI	The Children's Report of Parental Behavior Inventory	Schaefer, 1965
DAPCS	Dependency-Oriented and Achievement-Oriented Psychological Control Scale	Soenens et al., 2010
DASS	Depression Anxiety Stress Scales	Lovibond and Lovibond, 1995; Antony et al., 1998
DEAQ-A	The Depressive Experiences Questionnaire: Adolescents Version	Blatt et al., 1992
DSM-III-R	Diagnostic Interview for DSM-III	Silove et al., 1991
Family discussion task	Families were asked to discuss each of three situations by producing possible interpretations and solutions to the scenario	Luis et al., 2008
FEE	Recalled Parental Rearing Behavior	Schumacher et al., 1999
GAD	Generalized Anxiety Disorder Scale	Kroenke et al., 2007
HADS	Hospital Anxiety and Depression Scale	Zigmond and Snaith, 1983
HPBM	Helicopter Parenting Behaviors Measure	Schiffrin et al., 2014
HPBQ	Helicopter Parenting Behaviors Questionnaire	Schiffrin et al., 2014
HPQ	Helicopter Parenting Questionnaire	Padilla-Walker and Nelson, 2012
HPS	The Helicopter Parenting Scale	LeMoyne and Buchanan, 2011
IDAS	Inventory for Depression and Anxiety Symptoms	Watson et al., 2007
IDS	Inventory of Depressive Symptomatology	Rush et al., 1986
IPIP	International Personality Item Pool (subscales of depression and anxiety)	Goldberg et al., 2006
KDS	Kandel Depression Scale	Kandel and Davies, 1982
M-CIDI	Munich- Composite International Diagnostic Interview	Wittchen et al., 1998
MFQ-C	Child Mood and Feelings Questionnaire	Costello and Angold, 1988
MPSS	Mother's Parenting Style Scale	Oh and Lee, 1982
OHPQ	Original Helicopter Parenting Questionnaire	Schiffrin et al., 2014
OPS	Over Parenting Scale	Bradley-Geist and Olson-Buchanan, 2014
PBI	Parental bonding Instrument	Parker et al., 1979
PBI^*^	Parental Behavior Inventory	Barber, 1996
PCAGS	Psychological Control and Autonomy Scale	Barber, 1996
PCS	The Parental Control Scale	Padilla-Walker, 2008
PCS-YRS	Psychological Control Scale-Youth Self-Report	Barber, 1996
PCQ	Parenting Context Questionnaire	Grolnick and Wellborn, 1988
PHQ	Patient Health Questionnaire	Löwe et al., 2004
P-PASS	Perceived Parental Autonomy Support Scale	Bureau and Mageau, 2014
PPC	Psychological Control and Autonomy Granting Scale	Silk et al., 2003
PPK-TC: GELOPH-TC	Pho-Phi-Kat-Traditional Chinese Version: Geliophobia Subscale	Ruch and Proyer, 2009; Chen et al., 2011
PSI	Parenting Style index	Lamborn et al., 1991; Steinberg et al., 1994
RCMAS	Revised Children's Manifest Anxiety Scale	Reynolds and Richmond, 1978
SASA	Social Anxiety Scale for Adolescents	La Greca and Lopez, 1998
SCAI	Spence Child Anxiety Inventory	Spence, 1998
STAI	State Trait Anxiety Inventory	Spielberger et al., 1983
WMH-CIDI	Composite International Diagnostic Interview	Kessler and Üstün, 2004
YSR	Youth-Self-Report	Achenbach, 1991

The * symbol used to differentiate the measurements having same initials.

**Parenting style examined:** The studies focused on different parenting styles, see Table 4, for details. While 14 studies investigated helicopter parenting as an independent construct, 19 studies focused on the controlling dimension of helicopter parenting and 4 on the overprotective dimension. Additionally, 1 study investigated both the controlling and overprotective dimension. It should be noted that of the studies investigating a controlling parenting style, 3 focused solely upon maternal control (see Table 4). A total amount of 27 studies measured anxiety and 27 measured depression (see Table 4).

**Table 4:** Overview of parenting style and outcome examined as well as main findings.

**References**	**Parenting style**	**Outcome: anxiety**	**Outcome: depression**	**Main findings**
Basili et al. (2021)	Controlling parenting	X	X	Higher levels of parental control were associated with lower levels of anxiety and depression.
Cai and Tu (2020)	Controlling parenting * maternal control	X	X	No relationship between anxiety and controlling parenting. Higher levels of controlling parenting at T1 predicted higher levels of depressive symptoms at T2.
Cho et al. (2020)	Controlling parenting * maternal control	–	X	Lack of autonomy due to maternal control was associated with depressive symptoms
Cui et al. (2014)	Controlling parenting	–	X	Relationship between parental control and depressive symptoms
Cui et al. (2019)	Helicopter parenting	X	X	Helicopter parenting was associated with students' anxiety and depression level
Darlow et al. (2017)	Helicopter parenting	X	X	Higher levels of helicopter parenting were associated with more symptoms of depression. Helicopter parenting did not predict anxiety
Finkelstein et al. (2001)	Controlling parenting	–	X	No relationship between parental control and depression
Finkenauer et al. (2005)	Controlling parenting	–	X	Viewing one's parent as restrictive and psychological controlling was associated with depression
Gargurevich and Soenens (2016)	Controlling parenting (DPC and APC)	–	X	Both forms of parental psychological control (DPC and APC) were related to depressive symptoms
Goger et al. (2020)	Controlling parenting	X	–	Those who reported higher levels of controlling parenting also reported higher levels of anxiety.
Hong and Cui (2019)	Helicopter parenting	X	X	Higher levels of reported helicopter parenting was associated with higher levels of reported anxiety and depression
Heider et al. (2008)	Overprotective parenting	X	–	With the exception of GAD, there were a relationship between overprotection and anxiety. Regardless of the variations between single countries a similar parenting pattern across the four anxiety disorders was found
Inguglia et al. (2016)	Controlling parenting (DPC, APC and PAS)	X	X	APC was directly related to higher levels of anxiety and depression
Klein et al. (2020)	Controlling parenting	X	X	Those who scored high on depression and anxiety recalled their parents as controlling
Knappe et al. (2012)	Overprotective parenting	X	–	Overprotection was associated with social phobia
Kouros et al. (2017)	Helicopter parenting	X	–	Higher levels of perceived helicopter parenting was related to higher levels of social anxiety
Kullberg et al. (2021)	Controlling parenting	X	X	Higher levels of controlling parenting was related to higher levels of reported anxiety and depression
Kullberg et al. (2020)	Controlling parenting	X	X	Adults with anxiety disorder reported heighten maternal control. No direct relationship between control and depression.
LeMoyne and Buchanan (2011)	Helicopter parenting	–	–	Measurement for helicopter parenting was supported
Levitt et al. (2020)	Controlling parenting	X	X	Controlling parenting was positively associated with reported anxiety and depression levels
Lieb et al. (2000)	Overprotective parenting	X	–	Parental overprotection was found to be associated with social phobia.
Luebbe et al. (2018)	Helicopter parenting	X	X	Higher scores of helicopter parenting was associated with greater levels of depressive and anxiety symptoms
Luis et al. (2008)	Controlling parenting	X	–	Controlling parenting was associated with more anxiety
Mandara and Pikes (2008)	Controlling parenting * Maternal control	–	X	Controlling parenting was associated with higher depressive symptoms
Moilanen and Lynn Manuel (2019)	Helicopter parenting	–	X	High helicopter parenting was linked to high depression
Overbeek et al. (2007)	Overprotective parenting	X	X	Fathers' overprotection was significantly related to social phobia, and mothers' overprotection was significantly related to social and simple phobia. Maternal overprotection was found to be related to the subsequent onset of major depressive disorder
Reed et al. (2016)	Helicopter parenting	X	X	No direct effect of helicopter parenting and anxiety and depression
Reilly and Semkovska (2018)	Helicopter parenting	–	X	Perceived helicopter parenting predicted severity of depressive symptoms.
Reitman and Asseff (2010)	Controlling parenting	X	–	Perceptions of controlling parenting increased the reported anxiety
Rogers et al. (2020)	Controlling parenting	X	X	Adolescents who reported a relatively elevated and stable level of controlling parenting reported higher levels of anxiety and depression
Schiffrin et al. (2019)	Helicopter parenting	X	X	Perceived helicopter parenting increased self-reported symptoms of depression and anxiety
Schiffrin et al. (2014)	Helicopter parenting	X	X	Students who reported helicopter parenting also reported higher levels of depression. No effect for anxiety
Segrin et al. (2013)	Helicopter parenting	X	–	No direct effect of helicopter parenting on anxiety levels
Silove et al. (1991)	Controlling parenting	X	–	Those whom were diagnosed with GAD had a higher risk of perceiving their parents as someone who uses “affectionless control.” Those whom were diagnosed with PD were more likely to report “affectionate constraint”
Soenens et al. (2012)	Controlling parenting (DPC and APC)	–	X	Relationships between the domains of psychological control, depressive personality and depressive symptoms were found in both the Korean and Belgian group
Turner et al. (2020)	Helicopter parenting	–	X	A relationship between helicopter parenting and depression levels was detected
Wenze et al. (2019)	Helicopter parenting	X	X	Helicopter parenting was not directly related to depression or anxiety symptoms
Wu et al. (2018)	Overprotective and controlling parenting	X	–	Over controlling and overprotective parenting increased children's geliophobia.

### Anxiety

**Helicopter parenting and anxiety:** 10 studies examined the relationship between helicopter parenting and anxiety. Helicopter parenting was found to have a direct relationship with anxiety in 5 of the studies (Kouros et al., 2017; Luebbe et al., 2018; Cui et al., 2019; Hong and Cui, 2019; Schiffrin et al., 2019). However, in the remaining 5 studies there was no direct relationship between helicopter parenting and anxiety (Segrin et al., 2013; Schiffrin et al., 2014; Reed et al., 2016; Darlow et al., 2017; Wenze et al., 2019).

**Controlling parenting and anxiety:** A direct effect of controlling parenting with regard to increased anxiety was found in 11 of 12 studies that investigated the relationship between parental control and anxiety (Silove et al., 1991; Luis et al., 2008; Reitman and Asseff, 2010; Inguglia et al., 2016; Goger et al., 2020; Klein et al., 2020; Kullberg et al., 2020, 2021; Levitt et al., 2020; Rogers et al., 2020; Basili et al., 2021), whilst 1 did not (Cai and Tu, 2020). The only study that focused solely on maternal control and the outcome of anxiety did not find a relationship between the two variables (Cai and Tu, 2020).

**Overprotective parenting and anxiety:** No studies found reduced anxiety following overprotective parenting (Lieb et al., 2000; Overbeek et al., 2007; Heider et al., 2008; Knappe et al., 2012).

Overprotective and controlling parenting and anxiety: Lastly, there were 1 study that examined both the controlling and overprotective dimension of helicopter parenting simultaneously. This study found a relationship with anxiety (Wu et al., 2018).

### Depression

**Helicopter parenting and depression:** Nine of 11 studies investigating the relationship between helicopter parenting and depression, found an effect (Schiffrin et al., 2014, 2019; Darlow et al., 2017; Luebbe et al., 2018; Reilly and Semkovska, 2018; Cui et al., 2019; Hong and Cui, 2019; Moilanen and Lynn Manuel, 2019; Turner et al., 2020), whilst 2 did not (Reed et al., 2016; Wenze et al., 2019).

**Controlling parenting and depression:** 13 of 15 studies found a relationship between parental control and depression (Finkenauer et al., 2005; Mandara and Pikes, 2008; Soenens et al., 2012; Cui et al., 2014; Gargurevich and Soenens, 2016; Inguglia et al., 2016; Cai and Tu, 2020; Cho et al., 2020; Klein et al., 2020; Levitt et al., 2020; Rogers et al., 2020; Basili et al., 2021; Kullberg et al., 2021). The remaining 2 studies did not find a relationship (Finkelstein et al., 2001; Kullberg et al., 2020). In addition, 2 studies focused solely on maternal control, and both found a relationship with depression (Mandara and Pikes, 2008; Cho et al., 2020).

**Overprotective parenting and depression:** Overbeek et al. (2007) found a relationship between overprotective parenting and depression.

### Validity Assessment

Each individual study was assessed in accordance with Campbell's Validity Typology considering internal validity, statistical conclusion validity, construct validity as well as external validity (Shadish et al., 2002). Campbell's Validity Typology was chosen as it allows for more a more flexible validity assessment than that of other formal frameworks. This flexibility is particularly useful in a narrative synthesis such as this. A full overview of the validity assessment has been provided as Supplementary Materials. The basic assumption is that there is a casual link between helicopter parenting and the child outcomes of anxiety and depression. To establish this relationship one must examine whether the cause preceded the effect and if they are related, i.e., they covary (Shadish et al., 2002). Additionally, there cannot be any plausible alternative explanations for the effect. Thus, to investigate the relationship between helicopter parenting and anxiety and depression sufficiently, a longitudinal study, with validated measurements, must be conducted, as cross-sectional designs cannot establish the direction of the causal relationship. Out of the 38 included studies only 5 studies were longitudinal with 3 finding a relationship between parenting style and anxiety (Overbeek et al., 2007; Knappe et al., 2012; Rogers et al., 2020) and 2 finding a relationship with both and anxiety and depression (Overbeek et al., 2007; Rogers et al., 2020). Of the longitudinal studies 2 of them found no relationship with anxiety but did detect a relationship between parenting style and depression (Cai and Tu, 2020; Basili et al., 2021).

When evaluating the validity of each of these studies several risks of bias and validity threats were detected, which shall be mentioned briefly. Three studies only used child self-report, thereby exposing their study to the risk of mono-method bias and response bias (Overbeek et al., 2007; Knappe et al., 2012; Rogers et al., 2020). Cai and Tu (2020) only tested adolescent boys and their mothers, thus, their findings cannot be seen in context to family dynamic as a whole. One of the longitudinal studies, in particular, may be vulnerable to inflated risk of type 1 error due to multiple comparisons as they included 11 formal diagnoses for mental disorders in their analysis (Overbeek et al., 2007). Additionally, the parenting behavior only predicted the variance in mental health by a small amount (Overbeek et al., 2007). Regarding the study by Basili et al. (2021) the findings must be interpreted with caution as they found parental control to predict lower levels of depression, a surprising finding not supported by other studies. The assessment of each individual study can be found in more detail the Supplementary Materials provided.

Through this quality assessment, the study by Rogers et al. (2020) emerged as one of the strongest in terms of validity. This study takes into consideration the whole family dynamic in relation to controlling parenting and the potential outcome of anxiety and depression. Instead of only considering maternal, paternal, or not specifying which parent to keep in mind, these 500 adolescents reported on experienced maternal and parental control separately consistently from the age of 12 to 19. Thus, offering a deeper insight in the changing dynamics in the family across adolescence. The study found the majority of adolescents to report low but increasing levels of parent's psychological control across their second decade. The minority, however, who experience a stable and elevated level of controlling parenting also reported higher levels of symptoms of anxiety and depression. Cohen's d was calculated for maternal and paternal control to estimate the effect size on anxiety and depression. The effect size for both maternal control (.34) and parental control (.06) were found to be small in relation to reported depression. However, the effect size of maternal control (1.05) and parental control (0.8) were big in terms of reported adolescent anxiety.

## Discussion

The aim of this systematic review was to investigate helicopter parenting and its relationship with symptoms of anxiety and depression. Since helicopter parenting is a fairly new construct, this review considered parental control and overprotective parenting to be dimensions of helicopter parenting and thus, eligible for the study. The studies included in the review focused mostly on parental control, helicopter parenting and overprotective parenting, respectively. The majority of the studies revealed a relationship between helicopter parenting and anxiety and/or depression. However, issues with the validity of the findings were found and thus precluding firm conclusions regarding putative causal relationships as well as highlighting problems with self-report measurements of helicopter parenting.

A clear majority of the studies included in this review found a direct positive relationship between helicopter parenting and symptoms of anxiety and depression, with one exception (Basili et al., 2021) finding a negative association. Thus, the bulk of the studies suggest that parents behaving in an overprotective and controlling manner negatively affect their child's mental health. This relationship was detected in both adolescents and adults. Hence, helicopter parenting could have lifelong effects on an individual's anxiety and depression levels. Helicopter parenting was found to have a relationship with anxiety and/or depression in the US, Europe, Asia as well as South America, suggesting that this relationship goes beyond ethnicity and culture and supports the notion of helicopter parenting being a global phenomenon (Anderson, 2019). The implications of this research is vast. The findings of this systematic review have closed a gap in the research literature and provided future researchers with an overview of the evidence for the relationship between helicopter parenting and anxiety and depression. In addition, the findings of this review have detected helicopter parenting as a modifiable risk factor for anxiety and depression. It could be of use in the development of parenting interventions or to give health care professionals working with struggling families an insight in the nature of how a helicopter parenting can affect a child's mental health.

However, there are underlying issues with the validity of the studies included in this review. Firstly, one cannot establish a causal relationship unless it can be determined that the cause preceded the effect (Shadish et al., 2002). If we are to know that helicopter parenting causes anxiety or depression, we must determine that the helicopter parenting started before the manifestation of anxiety and depression. Although, the majority of the studies included in this review found a relationship with either anxiety or depression, most of the studies were cross-sectional. The cross-sectional study design is unable to address the direction of the effect (Shadish et al., 2002), hence internal validity is low. We can therefore not know with certainty if anxiety and depression causes helicopter parenting or if anxiety and depression in a child causes parents to act in lines with the helicopter parenting style. If we are to fully elucidate this relationship we need to employ a longitudinal study design. Out of few the longitudinal studies in this review all detected a relationship between helicopter parenting and either anxiety, depression or both.

Secondly, there is the issue of how helicopter parenting is measured. The most employed measures were subjective self-report measures, either from the parent themselves or from the child. The validity of parental self-report has been questioned (Holden, 2001; Locke and Prinz, 2002; Perepletchikova and Kazdin, 2004). In general, self-report measures are prone to bias and any results should be interpreted tentatively (Shadish et al., 2002). Self-report measures are dependent on the honesty of the respondents, but even if every respondent aimed to answer every item honestly, their introspective abilities could be lacking or they could be affected by common cognitive biases. People have been found to interpret and use the scales presented to them differently possibly producing different scores between participants reflecting something other than what the questionnaire was created to measure (Austin et al., 1998). Maybe especially so in anxiety research as people whom are high in anxiety have a tendency to be more extreme in their responses utilizing the endpoints of the scale more compared to those who hang around the midpoints (Lewis and Taylor, 1955; Austin et al., 1998). Despite this, several of the studies in this review chose to utilize self-report as the only measurement for all their variables. Thus, possibly causing monomethod bias where by the presenting everything in the same manner to the respondent could influence the results in and of itself (Shadish et al., 2002).

Additionally, parenting constructs have particular elements that are vulnerable to the distortion inherent in self-report (Morsbach and Prinz, 2006). Many parenting items can be considered highly sensitive in nature which strengthens the chance that parents attempt to present their own parenting in a way that are more socially desirable rather than choosing the response reflective of their true behavior (Tourangeau et al., 2000). It is a cognitively difficult task to make estimates of potentially high-frequency behavior, such as conversations had with child, over longer periods of time which in turn could lead to less precise estimations of their behavior (Tourangeau et al., 2000). There is also an uncertainty to the degree of consensus in the general population about the interpretations of certain parenting items, such as time-out (Clayman and Wissow, 2004). These factors might be why parents have been found to recall their parenting practice in lines of expert recommendations, even though it is inaccurate for their own practices (Robbins, 1963).

Following the inaccuracy parental self-report has on reality one could argue that it might be better to have the child report on the parenting style of their parents by retrospective recall. However, our memories are not accurate snapshots of the past, rather, they are constructions and must be understood as open to influence from the attempts to provide meaning (Schacter et al., 2011) and from the cognitive processes involving our selection and interpretation of elements making up memories (Chess et al., 1966). The validity of retrospective recall on the more subtle aspect of family life and relationships have been found to be less than satisfactory (Taylor and Brown, 1988). This might be because there are some evidence that what people remember could be influenced by their mood state at the time of the retrospective reporting (Bower, 1981; Matt et al., 1992). For instance, depressive mood fosters the recall of unhappy memories whilst inhibiting the recall of happy ones (Bower, 1981; Teasdale, 1983).

The concept of necessary and sufficient conditions for causal inferences can be applied in the endeavor to understand how helicopter parenting is related to anxiety and depression (Brennan, 2017). Research is clear that there are several reasons why one could develop depression, such as faulty mood regulation by the brain, genetic vulnerability and stressful life events (Burns et al., 2002; Bar, 2009). In terms of the causes of anxiety both genetic (Meier et al., 2019), the personality of the individual (Prince et al., 2021) as well as stressful life events could cause anxiety (Miloyan et al., 2018). The answer to the question of necessity is therefore, no: one need not necessarily be exposed to helicopter parenting to be depressed or anxious. Regarding whether helicopter parenting is a sufficient condition to develop anxiety and depression, this systematic review cannot determine that it is on an individual level. However, since most of the studies did detect a relationship, we cannot rule out the chance that helicopter parenting is a sufficient condition for anxiety and depression but an INUS condition according to Mackie (1965). INUS condition stands for *insufficient, but necessary part of an unnecessary but sufficient condition*. Mackie (1965) argued that effects, typically, have a plurality of conditional causes. Applying this philosophy to the topic at hand one could say that helicopter parenting could be a INUS condition for those who are already more vulnerable to mood disorders. It might also be that on a societal level, helicopter parenting, is sufficient in creating an environment characterized by heightened focus on dangers and uncertainties that might lead to increased population anxiety and depression.

Despite the issues of validity encountered in the endeavor to answer the question of helicopter parentings effect on anxiety and depression, Rogers et al. (2020) does a good job of it. This longitudinal study found there to be a relationship between controlling parenting and anxiety and depression. However, there were differences in the effect sizes in terms on how each specific parent effected anxiety and depression with maternal control found to have a bigger effect size than parental control. Additionally, although the effect size for both maternal and parental control was found to be quite small regarding its effect on depression, it was much bigger in terms of adolescent anxiety. These findings underline the utmost importance of including the whole family dynamic in future research. It could also be of interest to examine the different expectations adolescents have of their mothers and fathers in the transition to autonomy over their own lives.

One could argue that the question of the nature of the relationship between helicopter parenting and anxiety and depression is still answered somewhat unsatisfactorily. Nevertheless, the paramount importance of understanding it remains. Future research should therefore aim to develop longitudinal studies with higher validity where researchers thoroughly account for other factors that could possibly lead to the manifestation of anxiety and depression. To reduce the chance of bias the methods employed should not be limited to self-report, but also the observation of the actual parenting style, coded by researchers blinded to the hypothesis of the study to reduce researcher expectancy. It would also be helpful for future studies to take possible protective factors, such as self-efficacy, into account to better understand the relationship between helicopter parenting and anxiety and depression.

This review is not without its own limitations. There was only one reviewer undertaking the electronic literature search, consequently, it was decided that only two databases should be searched for eligible studies. It might be that there are some studies covering the topic of helicopter parenting and anxiety and depression in other relevant databases that was not found through this literature search or through scouring the reference lists of the included studies. In addition, there was only one reviewer conducting the initial screening procedure. Even though a second reviewer was contacted when uncertainty arose and the two reviews met to collectively go through the potential studies, it is still a recommendation by PRISMA that at least two separate screenings take place (Liberati et al., 2009). Akin to the other limitations mentioned, the initial quality assessment was completed by one reviewer before it was later discussed with a second reviewer. In addition, this systematic review did not investigate possible age differences in the outcomes of anxiety and depression.

Most of the evidence points in the direction of there being a relationship between helicopter parenting and anxiety and depression. However, issues with validity such as the possible distortion of self-report measures and few robust longitudinal studies undermine most of these findings. Findings suggest that it is important to include both maternal and paternal parenting style in future studies as they could affect the outcome of anxiety and depression differently. In conclusion, even though many of the studies included in this systematic review found a direct effect between helicopter parenting and anxiety and depression, the evidence for the nature of this relationship is insufficient and must be investigated further.

## Data Availability Statement

The original contributions presented in the study are included in the article/Supplementary Materials, further inquiries can be directed to the corresponding author/s.

## Author Contributions

JV was the first reviewer and conducted the systematic literature review. The records identified were screened firstly by JV and then collectively with KB. JV extracted the data and did the analysis. The findings of the analysis and the quality assessment was discussed with KB to reach a consensus. JV wrote the article, with the guidance of KB. All authors contributed to the article and approved the submitted version.

## Funding

Western University of Applied Sciences funded the open access publication fee.

## Conflict of Interest

The authors declare that the research was conducted in the absence of any commercial or financial relationships that could be construed as a potential conflict of interest.

## Publisher's Note

All claims expressed in this article are solely those of the authors and do not necessarily represent those of their affiliated organizations, or those of the publisher, the editors and the reviewers. Any product that may be evaluated in this article, or claim that may be made by its manufacturer, is not guaranteed or endorsed by the publisher.

## References

[B1] Achenbach T. M. (1991). Manual for the Youth Self-Report and 1991 Profile. Burlington: Department of Psychiatry.

[B2] Anderson J. (2019). The Much-Hated “Helicopter Parenting” Style Has Surprisingly Broad Appeal. Available online at: https://qz.com/1514079/much-hated-helicopter-parenting-style-is-surprisingly-popular/

[B3] Antony M. M. Bieling P. J. Cox B. J. Enns M. W. Swinson R. P. (1998). Psychometric properties of the 42-item and 21-item versions of the Depression Anxiety Stress Scales in clinical groups and a community sample. Psychol. Assess. 10, 176–181. 10.1037/1040-3590.10.2.176

[B4] Austin E. J. Deary I. J. Gibson G. J. McGregor M. J. Dent J. B. (1998). Individual response spread in self-report scales: personality correlations and consequences. Pers. Individ. Dif. 24, 421–438. 10.1016/S0191-8869(97)00175-X

[B5] Bandura A. (2010). Self-efficacy the Corsini Encyclopedia of Psychology. John Wiley & Sons, Inc. 10.1002/9780470479216.corpsy0836

[B6] Bandura A. Pastorelli C. Barbaranelli C. Caprara G. V. (1999). Self-efficacy pathways to childhood depression. J. Pers. Soc. Psychol. 76, 258. 10074708 10.1037//0022-3514.76.2.258

[B7] Bar M. (2009). A cognitive neuroscience hypothesis of mood and depression. Trends Cogn. Sci. 13, 456–463. 10.1016/j.tics.2009.08.009 19819753 PMC2767460

[B8] Barber B. K. (1996). Parental psychological control: revisiting a neglected construct. Child Dev. 67, 3296–3319. 10.2307/1131780 9071782

[B9] Barber B. K. Olsen J. E. Shagle S. C. (1994). Associations between parental psychological and behavioral control and youth internalized and externalized behaviors. Child Dev. 65, 1120–1136. 10.2307/1131309 7956469

[B10] Basili E. Zuffian,ò A. Pastorelli C. Thartori E. Lunetti C. Favini A. . (2021). Maternal and paternal psychological control and adolescents' negative adjustment: a dyadic longitudinal study in three countries. PLoS ONE 16, e0251437. 10.1371/journal.pone.0251437 33989323 PMC8121295

[B11] Beck A. T. Epstein N. Brown G. Steer R. A. (1988). An inventory for measuring clinical anxiety: psychometric properties. J. Consult. Clin. Psychol. 56, 893. 10.1037/0022-006X.56.6.893 3204199

[B12] Beck A. T. Steer R. A. Brown G. (1996). Beck depression inventory–II. Psychol. Assess. 56, 893–897. 10.1037/t00742-000

[B13] Belsky J. (1984). The determinants of parenting: a process model. Child Dev. 83–96. 10.2307/1129836 6705636

[B14] Blatt S. J. Schaffer C. E. Bers S. A. Quinlan D. M. (1992). Psychometric properties of the depressive experiences questionnaire for adolescents. J. Pers. Assess. 59, 82–98. 10.1207/s15327752jpa5901_8 1512682

[B15] Bower G. H. (1981). Mood and memory. Am. Psychol. 36, 129. 10.1037/0003-066X.36.2.129 7224324

[B16] Bradley-Geist J. C. Olson-Buchanan J. B. (2014). Helicopter parents: an examination of the correlates of over-parenting of college students. Educ. Train. 54, 314–328. 10.1108/ET-10-2012-0096

[B17] Brennan A. (2017, May 18). Necessary and sufficient conditions. Stanford Encyclopedia of Philosophy. Available online at: https://plato.stanford.edu/entries/necessary-sufficient/

[B18] Bureau J. S. Mageau G. A. (2014). Parental autonomy support and honesty: the mediating role of identification with the honesty value and perceived costs and benefits of honesty. J. Adolesc. 37, 225–236. 10.1016/j.adolescence.2013.12.007 24636683

[B19] Burns J. M. Andrews G. Szabo M. (2002). Depression in young people: what causes it and can we prevent it? Med. J. Aust. 177, S93–S96. 10.5694/j.1326-5377.2002.tb04864.x 12358564

[B20] Cai T. Tu K. M. (2020). Linking parental monitoring and psychological control with internalizing symptoms in early adolescence: the moderating role of vagal tone. J. Abnorm. Child Psychol. 48, 809–821. 10.1007/s10802-020-00631-w 32170524

[B21] Chen H. C. Chan Y. C. Ruch W. Proyer R. T. (2011). Evaluating the reliability and validity of a traditional Chinese version of the PhoPhiKat-45. Psychol. Test. 58, 119–145.

[B22] Chess S. Thomas A. Birch H. G. (1966). Distortions in developmental reporting made by parents of behaviorally disturbed children. J. Am. Acad. Child Psychiatry. 5, 226–234. 10.1016/S0002-7138(09)62054-9 5908286

[B23] Cho J. Ha J. H. Jue J. (2020). Influences of the differences between mothers' and children's perceptions of parenting styles. Front. Psychol. 11, 552585. 10.3389/fpsyg.2020.552585 33192802 PMC7653220

[B24] Clayman M. L. Wissow L. S. (2004). Pediatric residents' response to ambiguous words about child discipline and behavior. Patient Educ. Couns. 55, 16–21. 10.1016/S0738-3991(03)00245-3 15476985

[B25] Cline F. Fay J. (2020). Parenting with Love and Logic: Teaching Children Responsibility. Colorado Springs: NavPress Publishing Group.

[B26] Costello E. J. Angold A. (1988). Scales to assess child and adolescent depression: checklists, screens, and nets. J. Am. Acad. Child Adolescent Psychiatry 27, 726–737. 10.1097/00004583-198811000-00011 3058677

[B27] Cui L. Morris A. S. Criss M. M. Houltberg B. J. Silk J. S. (2014). Parental psychological control and adolescent adjustment: the role of adolescent emotion regulation. Parenting 14, 47–67. 10.1080/15295192.2014.880018 25057264 PMC4104177

[B28] Cui M. Janhonen-Abruquah H. Darling C. A. Chavez F. L. Palojoki P. (2019). Helicopter parenting and young adults' well-being: a comparison between United States and Finland. Cross Cultural Res. J. Compar. Soc. Sci. 53, 410–427. 10.1177/1069397118802253

[B29] Darling N. Steinberg L. (1993). Parenting style as context: an integrative model. Psychol. Bull. 113, 487–496. 10.1037/0033-2909.113.3.487

[B30] Darlow V. Norvilitis J. M. Schuetze P. (2017). The relationship between helicopter parenting and adjustment to college. J. Child Fam. Stud. 26, 2291–2298. 10.1007/s10826-017-0751-330369779

[B31] Finkelstein J.-A. Donenberg G. Martinovich Z. (2001). Maternal control and adolescent depression: ethnic differences among clinically referred girls. Multidisciplinary Res. Publ. 30, 155–171. 10.1023/A:1010341724157

[B32] Finkenauer C. Engels R. C. M. E. Baumeister R. F. (2005). Parenting behaviour and adolescent behavioural and emotional problems: the role of self-control. Int. J. Behav. Dev. 29, 58–69. 10.1080/01650250444000333

[B33] Gargurevich R. Soenens B. (2016). Psychologically controlling parenting and personality vulnerability to depression: a study in Peruvian late adolescents. J. Child Fam. Stud. 25, 911–921. 10.1007/s10826-015-0265-9

[B34] Goger P. Rozenman M. Gonzalez A. (2020). The association between current maternal psychological control, anxiety symptoms, and emotional regulatory processes in emerging adults. J. Behav. Ther. Exp. Psychiatry 68, 101563. 10.1016/j.jbtep.2020.101563 32145580 PMC7214129

[B35] Goldberg L. R. Johnson J. A. Eber H. W. Hogan R. Ashton M. C. Cloninger C. R. . (2006). The International Personality Item Pool and the future of public-domain personality measures. J. Res. Pers. 40, 84–96. 10.1016/j.jrp.2005.08.007

[B36] Gray P. (2015). Declining student resilience: a serious problem for colleges. Psychol. Today 22, 9–22.

[B37] Grolnick W. Wellborn J. (1988). “Parent influences on children's school-related self-system process,” in Annual Meeting of the American Educational Research Association (New Orleans, LA).

[B38] Gutman L. M. Feinstein L. (2010). Parenting behaviours and children's development from infancy to early childhood: changes, continuities and contributions. Early Child Dev. Care 180, 535–556. 10.1080/03004430802113042

[B39] Haidt J. Lukianoff G. (2018). The Coddling of the American Mind: How Good Intentions and Bad Ideas Are Setting Up a Generation for Failure. New York, NY: Penguin UK.

[B40] Heider D. Matschinger H. Bernert S. Alonso J. Brugha T. Bruffaerts R. . (2008). Adverse parenting as a risk factor in the occurrence of anxiety disorders. Int. J. Res. Soc. Genetic Epidemiol. Mental Health Serv. 43, 266–272. 10.1007/s00127-007-0302-0 18196186

[B41] Holden G. W. (2001). “Parenthood,” in Handbook of Family Measurement Techniques, Vol. 1, eds J. Touliatos, Perlmutter B. F., and M. A. Straus (California: Sage), 137–149.

[B42] Hong P. Cui M. (2019). Helicopter parenting and college students' psychological maladjustment: the role of self-control and living arrangement. J. Child Fam. Stud. 29, 338–347. 10.1007/s10826-019-01541-2

[B43] Inguglia C. Ingoglia S. Liga F. Lo Coco A. Lo Cricchio M. Musso P. . (2016). Parenting dimensions and internalizing difficulties in Italian and U.S. emerging adults: the intervening role of autonomy and relatedness. J. Child Fam. Stud. 25, 419–431. 10.1007/s10826-015-0228-1

[B44] Ishizuka P. (2019). Social class, gender, and contemporary parenting standards in the United States: evidence from a national survey experiment. Soc. Forces 98, 31–58. 10.1093/sf/soy107

[B45] Kandel D. B. Davies M. (1982). Epidemiology of depressive mood in adolescents: an empirical study. Arch. Gen. Psychiatry 39, 1205–1212. 10.1001/archpsyc.1982.04290100065011 7125850

[B46] Kanfer R. Zeiss A. M. (1983). Depression, interpersonal standard setting, and judgments of self-efficacy. J. Abnorm. Psychol. 92, 319. 10.1037/0021-843X.92.3.319 6619407

[B47] Keijsers L. Poulin F. (2013). Developmental changes in parent–child communication throughout adolescence. Dev. Psychol. 49, 2301. 10.1037/a0032217 23477535

[B48] Kessler R. C. Üstün T. B. (2004). The world mental health (WMH) survey initiative version of the world health organization (WHO) composite international diagnostic interview (CIDI). Int. J. Methods Psychiatr. Res. 13, 93–121. 10.1002/mpr.168 15297906 PMC6878592

[B49] Klein E. M. Brähler E. Petrowski K. Tibubos A. N. Burghardt J. Wiltink J. . (2020). Recalled parental rearing behavior in adult women and men with depressive and anxiety symptoms: findings from a community study. Z. Psychosom. Med. Psychother. 66, 243–258. 10.13109/zptm.2020.66.3.243 32876552

[B50] Knappe S. Beesdo-Baum K. Fehm L. Lieb R. Wittchen H.-U. (2012). Characterizing the association between parenting and adolescent social phobia. J. Anxiety Disord. 26, 608–616. 10.1016/j.janxdis.2012.02.014 22445318

[B51] Kouros C. D. Pruitt M. M. Ekas N. V. Kiriaki R. Sunderland M. (2017). Helicopter parenting, autonomy support, and college students' mental health and well-being: the moderating role of sex and ethnicity. J. Child Fam. Stud. 26, 939–949. 10.1007/s10826-016-0614-3 31832009 PMC6907082

[B52] Kovacs M. Preiss M. (1992). CDI. Children's Depression Inventory. New York, NY: Multi-Health Systems.

[B53] Kroenke K. Spitzer R. L. Williams J. B. Monahan P. O. Löwe B. (2007). Anxiety disorders in primary care: prevalence, impairment, comorbidity, and detection. Ann. Intern. Med. 146, 317–325. 10.7326/0003-4819-146-5-200703060-00004 17339617

[B54] Kullberg M. J. van Schie C. C. van Sprang E. D. Hartman C. A. van Hemert A. M. Penninx B. . (2021). Why some siblings thrive whereas others struggle: a within-family study on recollections of childhood parental bonding and current adult depressive and anxiety symptoms. J. Affect. Disord. 281, 413–421. 10.1016/j.jad.2020.12.045 33359954

[B55] Kullberg M. L. Maciejewski D. van Schie C. C. Penninx B. Elzinga B. M. (2020). Parental bonding: psychometric properties and association with lifetime depression and anxiety disorders. Psychol. Assess. 32, 780–795. 10.1037/pas0000864 32463266

[B56] La Greca A. M. Lopez N. (1998). Social anxiety among adolescents: linkages with peer relations and friendships. J. Abnorm. Child Psychol. 26, 83–94. 10.1023/A:1022684520514 9634131

[B57] Lamborn S. D. Mounts N. S. Steinberg L. Dornbusch S. M. (1991). Patterns of competence and adjustment among adolescents from authoritative, authoritarian, indulgent, and neglectful families. Child Dev. 62, 1049–1065. 10.2307/1131151 1756655

[B58] LeMoyne T. Buchanan T. (2011). Does “hovering” matter? Helicopter parenting and its effect on well-being. Sociol. Spectrum 31, 399–418. 10.1080/02732173.2011.574038

[B59] Levitt M. R. Grolnick W. S. Caruso A. J. Lerner R. E. (2020). Internally and externally controlling parenting: relations with children's symptomatology and adjustment. J. Child Fam. Stud. 29, 3044–3058. 10.1007/s10826-020-01797-z

[B60] Lewis N. A. Taylor J. A. (1955). Anxiety and extreme response preferences. Educ. Psychol. Meas. 15, 111–116. 10.1177/001316445501500203

[B61] Liberati A. Altman D. G. Tetzlaff J. Mulrow C. Gøtzsche P. C. Ioannidis J. P. . (2009). The PRISMA statement for reporting systematic reviews and meta-analyses of studies that evaluate health care interventions: explanation and elaboration. J. Clin. Epidemiol. 62, e1–e34. 10.1016/j.jclinepi.2009.06.006 19631507

[B62] Lieb R. Wittchen H.-U. Höfler M. Fuetsch M. Stein M. B. Merikangas K. R. (2000). Parental psychopathology, parenting styles, and the risk of social phobia in offspring: a prospective-longitudinal community study. Arch. Gen. Psychiatry 57, 859–866. 10.1001/archpsyc.57.9.859 10986549

[B63] Locke L. M. Prinz R. J. (2002). Measurement of parental discipline and nurturance. Clin. Psychol. Rev. 22, 895–929. 10.1016/S0272-7358(02)00133-2 12214330

[B64] Lovibond S. H. Lovibond P. F. (1995). Manual for the Depression Anxiety Stress Scales, 2nd Edn. Oxford: Psychology Foundation of Australia.

[B65] Löwe B. Kroenke K. Herzog W. Gräfe K. (2004). Measuring depression outcome with a brief self-report instrument: sensitivity to change of the Patient Health Questionnaire (PHQ-9). J. Affect. Disord. 81, 61–66. 10.1016/S0165-0327(03)00198-8 15183601

[B66] Luebbe A. M. Mancini K. J. Kiel E. J. Spangler B. R. Semlak J. L. Fussner L. M. (2018). Dimensionality of helicopter parenting and relations to emotional, decision-making, and academic functioning in emerging adults. Assessment 25, 841–857. 10.1177/1073191116665907 27561986

[B67] Luis T. M. Varela R. E. Moore K. W. (2008). Parenting practices and childhood anxiety reporting in Mexican, Mexican American, and European American families. J. Anxiety Disord. 22, 1011–1020. 10.1016/j.janxdis.2007.11.001 18083326

[B68] Lythcott-Haims J. (2015). The Four Cultural Shifts That Led to the Rise of the Helicopter Parent. Available online at: https://www.businessinsider.com/the-rise-of-the-helicopter-parent-2015-7?r=USandIR=T

[B69] Mackie J. L. (1965). Causes and conditions. Am. Philos. Q. 2, 245–264.

[B70] Mandara J. Pikes C. L. (2008). Guilt trips and love withdrawal: does mothers? Use of psychological control predict depressive symptoms among African American adolescents?^*^. Fam. Relat. 57, 602–612. 10.1111/j.1741-3729.2008.00526.x

[B71] Masud H. Thurasamy R. Ahmad M. S. (2015). Parenting styles and academic achievement of young adolescents: a systematic literature review. Qual. Quantity 49, 2411–2433. 10.1007/s11135-014-0120-x

[B72] Matt G. E. Vázquez C. Campbell W. K. (1992). Mood-congruent recall of affectively toned stimuli: a meta-analytic review. Clin. Psychol. Rev. 12, 227–255. 10.1016/0272-7358(92)90116-P

[B73] Meier S. M. Trontti K. Purves K. L. Als T. D. Grove J. Laine M. . (2019). Genetic variants associated with anxiety and stress-related disorders: a genome-wide association study and mouse-model study. JAMA Psychiatry 76, 924–932. 10.1001/jamapsychiatry.2019.1119 31116379 PMC6537792

[B74] Miloyan B. Bienvenu O. J. Brilot B. Eaton W. W. (2018). Adverse life events and the onset of anxiety disorders. Psychiatry Res. 259, 488–492. 10.1016/j.psychres.2017.11.027 29154170 PMC7831231

[B75] Moher D. Liberati A. Tetzlaff J. Altman D. G. (2010). Preferred reporting items for systematic reviews and meta-analyses: the PRISMA statement. Int. J. Surg. 8, 336–341. 10.1016/j.ijsu.2010.02.007 20171303

[B76] Moilanen K. L. Lynn Manuel M. (2019). Helicopter parenting and adjustment outcomes in young adulthood: a consideration of the mediating roles of mastery and self-regulation. J. Child Fam. Stud. 28, 2145–2158. 10.1007/s10826-019-01433-5

[B77] Morsbach S. Prinz R. (2006). Understanding and improving the validity of self-report of parenting. Clin. Child Fam. Psychol. Rev. 9, 1–21. 10.1007/s10567-006-0001-5 16636897

[B78] Muris P. Schouten E. Meesters C. Gijsbers H. (2003). Contingency-competence-control? Related beliefs and symptoms of anxiety and depression in a young adolescent sample. Child Psychiatry Hum. Dev. 33, 325–339. 10.1023/A:1023040430308 12723904

[B79] Nutt E. A. (2018). Why Kids and Teens May Face far More Anxiety These Days. Available online at: https://www.washingtonpost.com/news/to-your-health/wp/2018/05/10/why-kids-and-teens-may-face-far-more-anxiety-these-days/?noredirect=onandutm_term=.96d024a6cbd6

[B80] Oh S. Lee J. (1982). The relationship between children's perception on and the affective characteristic of parental child-rearing style. Res. Note 11, 1–8.

[B81] Overbeek G. Have M. Vollebergh W. Graaf R. (2007). Parental lack of care and overprotection; longitudinal associations with DSM-III-R disorders.(Author abstract). Soc. Psychiatry Psychiatr. Epidemiol. 42, 87. 10.1007/s00127-006-0115-6 17195899

[B82] Padilla-Walker L. M. (2008). Domain-appropriateness of maternal discipline as a predictor of adolescents' positive and negative outcomes. J. Fam. Psychol. 22, 456–464. 10.1037/0893-3200.22.3.456 18540774

[B83] Padilla-Walker L. M. Nelson L. J. (2012). Black hawk down?: Establishing helicopter parenting as a distinct construct from other forms of parental control during emerging adulthood. J. Adolesc. 35, 1177–1190. 10.1016/j.adolescence.2012.03.007 22503075

[B84] Parker G. Tupling H. Brown L. B. (1979). Parental bonding instrument (PBI). Br. J. Med. Psychol. 52, 1–10. 10.1111/j.2044-8341.1979.tb02487.x

[B85] Perepletchikova F. Kazdin A. E. (2004). Assessment of parenting practices related to conduct problems: development and validation of the Management of Children's Behavior Scale. J. Child Fam. Stud. 13, 385–403. 10.1023/B:JCFS.0000044723.45902.70

[B86] Perry N. B. Dollar J. M. Calkins S. D. Keane S. P. Shanahan L. (2018). Childhood self-regulation as a mechanism through which early overcontrolling parenting is associated with adjustment in preadolescence. Dev. Psychol. 54, 1542. 10.1037/dev0000536 29911876 PMC6062452

[B87] Prince E. J. Siegel D. J. Carroll C. P. Sher K. J. Bienvenu O. J. (2021). A longitudinal study of personality traits, anxiety, and depressive disorders in young adults. Anxiety Stress Coping 34, 299–307. 10.1080/10615806.2020.1845431 33190525 PMC8068574

[B88] Radloff L. S. (1977). The CES-D scale: a self-report depression scale for research in the general population. Appl. Psychol. Meas. 1, 385–401. 10.1177/01466216770010030623302475

[B89] Reed K. Duncan J. M. Lucier-Greer M. Fixelle C. Ferraro A. J. (2016). Helicopter parenting and emerging adult self-efficacy: implications for mental and physical health. J. Child Fam. Stud. 25, 3136–3149. 10.1007/s10826-016-0466-x

[B90] Reilly S. Semkovska M. (2018). An examination of the mediatory role of resilience in the relationship between helicopter parenting and severity of depressive symptoms in Irish university students. Adolescent Psychiatry 8, 32–47. 10.2174/2210676608666180508130224

[B91] Reitman D. Asseff J. (2010). Parenting practices and their relation to anxiety in young adulthood. J. Anxiety Disord. 24, 565–572. 10.1016/j.janxdis.2010.03.016 20456909

[B92] Reynolds C. R. Richmond B. O. (1978). What I think and feel: a revised measure of children's manifest anxiety. J. Abnorm. Child Psychol. 6, 271–280. 10.1007/BF00919131 670592

[B93] Robbins L. C. (1963). The accuracy of parental recall of aspects of child development and of child rearing practices. J. Abnormal Soc. Psychol. 66, 261. 10.1037/h0049084 13974231

[B94] Rogers A. A. Padilla-Walker L. M. McLean R. D. Hurst J. L. (2020). Trajectories of perceived parental psychological control across adolescence and implications for the development of depressive and anxiety symptoms. J. Youth Adolesc. 49, 136–149. 10.1007/s10964-019-01070-7 31273602

[B95] Ruch W. Proyer R. T. (2009). Extending the studying of gelotophobia: on gelotophilies and katagelasticsits. Humor Int. J. Humor Res. 22, 183–212. 10.1515/HUMR.2009.009

[B96] Rush A. J. Giles D. E. Schlesser M. A. Fulton C. L. Weissenburger J. Burns C. (1986). The inventory for depressive symptomatology (IDS): preliminary findings. Psychiatry Res. 18, 65–87. 10.1016/0165-1781(86)90060-0 3737788

[B97] Ryan R. M. Deci E. L. (2000). Self-determination theory and the facilitation of intrinsic motivation, social development, and well-being. Am. Psychol. 55, 68. 10.1037/0003-066X.55.1.68 11392867

[B98] Santos C. M. da C. Pimenta C. A. de M. Nobre M. R. C. (2007). The PICO strategy for the research question construction and evidence search. Rev. Lat. Am. Enfermagem 15, 508–511. 10.1590/S0104-11692007000300023 17653438

[B99] Santrock J. W. (2019). Life-Span Development, 17th Edn. Columbus: McGraw-Hill Education.

[B100] Schacter D. L. Guerin S. A. Jacques P. L. S. (2011). Memory distortion: an adaptive perspective. Trends Cogn. Sci. 15, 467–474. 10.1016/j.tics.2011.08.004 21908231 PMC3183109

[B101] Schaefer E. S. (1965). Children's reports of parental behavior: an inventory. Child Dev. 36, 413–424. 10.2307/1126465 14300862

[B102] Schiffrin H. H. Erchull M. J. Sendrick E. Yost J. C. Power V. Saldanha E. R. (2019). The effects of maternal and paternal helicopter parenting on the self-determination and well-being of emerging adults. J. Child Fam. Stud. 28, 3346–3359. 10.1007/s10826-019-01513-6

[B103] Schiffrin H. H. Liss M. (2017). The effects of helicopter parenting on academic motivation. J. Child Fam. Stud. 26, 1472–1480. 10.1007/s10826-017-0658-z

[B104] Schiffrin H. H. Liss M. Miles-McLean H. Geary K. A. Erchull M. J. Tashner T. (2014). Helping or hovering? The effects of helicopter parenting on college students' well-being. J. Child Fam. Stud. 23, 548–557. 10.1007/s10826-013-9716-3

[B105] Schneider B. Klager C. Chen I.-C. Burns J. (2016). Transitioning into adulthood: striking a balance between support and independence. Policy Insights Behav. Brain Sci. 3, 106–113. 10.1177/2372732215624932

[B106] Schumacher J. Eisemann M. Brahler E. (1999). Looking back on parents: The Questionnaire of Recalled Parental Rearing Behaviour (QRPRB). Diagnostica 45, 194–204. 10.1026//0012-1924.45.4.194

[B107] Seery M. D. Holman E. A. Silver R. C. (2010). Whatever does not kill us: cumulative lifetime adversity, vulnerability, and resilience. J. Pers. Soc. Psychol. 99, 1025–1041. 10.1037/a0021344 20939649

[B108] Segrin C. Woszidlo A. Givertz M. Montgomery N. (2013). Parent and child traits associated with overparenting. J. Soc. Clin. Psychol. 32, 569–595. 10.1521/jscp.2013.32.6.569

[B109] Shadish W. R. Cook T. D. Campbell D. T. (eds.). (2002). Experimental and Quasi-Experimental Designs for Generalized Causal Inference. Boston, MA: Houghton Mifflin.

[B110] Silk J. S. Morris A. S. Kanaya T. Steinberg L. (2003). Psychological control and autonomy granting: opposite ends of a continuum or distinct constructs? J. Res. Adolescence 13, 113–128. 10.1111/1532-7795.1301004

[B111] Silove D. Parker G. Hadzi-Pavlovic D. Manicavasagar V. Blaszczynski A. (1991). Parental representations of patients with panic disorder and generalised anxiety disorder. Br. J. Psychiatry 159, 835–841. 10.1192/bjp.159.6.835 1790454

[B112] Smeets R. Dingemans P. (1993). Composite International Diagnostic Interview (CIDI), Version 1.1. Amsterdam; Geneva: World Health Organization.

[B113] Soenens B. Park S.-Y. Vansteenkiste M. Mouratidis A. (2012). Perceived parental psychological control and adolescent depressive experiences: a cross-cultural study with Belgian and South-Korean adolescents. J. Adolesc. 35, 261–272. 10.1016/j.adolescence.2011.05.001 21620464

[B114] Soenens B. Vansteenkiste M. Luyten P. (2010). Toward a domain-specific approach to the study of parental psychological control: distinguishing between dependency-oriented and achievement-oriented psychological control. J. Pers. 78, 217–256. 10.1111/j.1467-6494.2009.00614.x 20433618

[B115] Spence S. H. (1998). A measure of anxiety symptoms among children. Behav. Res. Ther. 36, 545–566. 10.1016/S0005-7967(98)00034-5 9648330

[B116] Spielberger C. D. Gorsuch R. L. Lushene R. Vagg P. R. Jacobs G. A. (1983). Manual for the State-Trait Anxiety Inventory. Redwood City: Consulting Psychologists Press.

[B117] Spokas M. Heimberg R. G. (2009). Overprotective parenting, social anxiety, and external locus of control: cross-sectional and longitudinal relationships. Cognit. Ther. Res. 33, 543. 10.1007/s10608-008-9227-5

[B118] Steinberg L. Lamborn S. D. Darling N. Mounts N. S. Dornbusch S. M. (1994). Over-time changes in adjustment and competence among adolescents from authoritative, authoritarian, indulgent, and neglectful families. Child Dev. 65, 754–770. 10.2307/1131416 8045165

[B119] Szwedo D. E. Hessel E. T. Loeb E. L. Hafen C. A. Allen J. P. (2017). Adolescent support seeking as a path to adult functional independence. Dev. Psychol. 53, 949–961. 10.1037/dev0000277 28358534 PMC5469408

[B120] Taylor S. E. Brown J. D. (1988). Illusion and well-being: a social psychological perspective on mental health. Psychol. Bull. 103, 193. 10.1037/0033-2909.103.2.193 3283814

[B121] Teachman B. A. Allen J. P. (2007). Development of social anxiety: social interaction predictors of implicit and explicit fear of negative evaluation. J. Abnorm. Child Psychol. 35, 63–78. 10.1007/s10802-006-9084-1 17171538 PMC3395171

[B122] Teasdale J. D. (1983). Negative thinking in depression: cause, effect, or reciprocal relationship? Adv. Behav. Res. Ther. 5, 3–25. 10.1016/0146-6402(83)90013-9

[B123] Tourangeau R. Rips L. J. Rasinski K. (2000). The Psychology of Survey Response. Cambridge University Press.

[B124] Turner L. A. Faulk R. D. Garner T. (2020). Helicopter parenting, authenticity, and depressive symptoms: a mediation model. J. Genetic Psychol. Res. Theory Human Dev. 181, 500–505. 10.1080/00221325.2020.1775170 32552440

[B125] Twenge J. M. Cooper A. B. Joiner T. E. Duffy M. E. Binau S. G. (2019). Age, period, and Cohort trends in mood disorder indicators and suicide-related outcomes in a nationally representative dataset, 2005-2017. J. Abnorm. Psychol. 128, 185–199. 10.1037/abn0000410 30869927

[B126] Waller R. Gardner F. Hyde L. W. (2013). What are the associations between parenting, callous–unemotional traits, and antisocial behavior in youth? A systematic review of evidence. Clin. Psychol. Rev. 33, 593–608. 10.1016/j.cpr.2013.03.001 23583974

[B127] Watson D. O'Hara M. W. Simms L. J. Kotov R. Chmielewski M. McDade-Montez E. A. . (2007). Development and validation of the Inventory of Depression and Anxiety Symptoms (IDAS). Psychol. Assess. 19, 253. 10.1037/1040-3590.19.3.253 17845118

[B128] Wenze S. J. Pohoryles A. B. DeCicco J. M. (2019). Helicopter parenting and emotion regulation in U.S. college students. Psi Chi J. Psychol. Res. 24, 274–283. 10.24839/2325-7342.JN24.4.210

[B129] Wittchen H. U. Lachner G. Wunderlich U Pfister H. (1998). Test-retest reliability of the computerized DSM-IV version of the Munich-Composite International Diagnostic Interview (M-CIDI). Soc. Psychiatry Psychiatr. Epidemiol. 33, 568–578. 10.1007/s001270050095 9803825

[B130] World Health Organization (2021). Adolescent Health. World Health Organization. Available online at: https://www.who.int/health-topics09/adolescent-health#tab=tab_1

[B131] Wu C.-L. Huang Y.-T. Wang P.-Y. Chen H.-C. (2018). Parent-child attachment as a mediator of the relationship between parenting style and gelotophobia among adolescents. Int. J. Psychol. 54, 548–556. 10.1002/ijop.12493 29931673

[B132] Zigmond A. S. Snaith R. P. (1983). The hospital anxiety and depression scale. Acta Psychiatr. Scand. 67, 361–370. 10.1111/j.1600-0447.1983.tb09716.x 6880820

