# Supplementary material for: A Systematic Review of “Helicopter Parenting” and Its Relationship With Anxiety and Depression
Source: Front Psychol. 2022 May 25;13:872981. doi: 10.3389/fpsyg.2022.872981 (PMC9176408; doi:10.3389/fpsyg.2022.872981)
Supplement: Supplementary file 1 [file Table_1.docx]

Supplementary Material

# Quality Assessment

| **Author(s) and year** | **Internal** | **External** | **Stat conclusion** | **Construct** |
| --- | --- | --- | --- | --- |
| Basili et al., 2020 | Cross-sectional data, cannot address the direction of effects.  Some completed the test at home and some at another location. Could have affected the baseline anxiety. | Groups were not equal in size. Italy n=148 US =154 Colombia n=74 | The unexpected negative effect between parental control and anxiety and depression in adolescents must be taken with caution as no other studies explicitly support this finding  Concern regards to the length of testing. Young adolescents could have become bored and not give accurate replies. Possibly measurement error. | Self-report. Response bias. |
| Cai & Tu, 2020 |  | Only tested adolescent boys and their mothers. Cannot be generalized to girls. Fathers could have different implications for adolescent mental health. Cannot be seen in context to family dynamic as a whole. |  | Self-report.   The only measurement for parenting was self-report by the mothers themselves. Possible response bias, |
| Cho et al., 2020 | Cross-sectional data. Cannot infer causation. Could be that adolescents whom struggles with depression causes mothers to be controlling.  Researcher was present during testing. Researcher expectancy bias.  Test conducted in class, could affect baseline anxiety. | Sample consisted of Korean mothers and their adolescent. Findings cannot be generalized as Korea is a highly competitive society with fierce academic competition.  Cannot be seen in context to family dynamic as a whole. Fathers could have a different impact on adolescent mental health. |  | Self-report. Response bias. |
| Cui et al 2014, | Cross-sectional data, cannot address the direction of effects. It might be adolescent behaviour problems that elicit more psychologically controlling strategies from parents. The indirect effects of psychological control through anger regulations should also be interpreted with caution.  There might be a ‘higher need’ to be protective in a low-income and disadvantage community than in an upper-class one, this was not measured for.  Reactivity to the experimental situation. Participants could have responded differently due to having to conduct the study at the university. Could have affected their base anxiety. | Subsample size of each ethnic group was small.  Participants recruited from disadvantaged communities with high percentage of ethnic minority and low-income families and can therefore not be generalized to another population. |  | Self-report. Response bias. Mono-method bias.  The emotional regulation measure taps into adolescents’ general abilities of emotion regulations and does not take context into account.  Internal consistency of parent- and adolescent-reported sadness regulation were low – parent report= .60 adolescent report= .61 This low internal consistency can give us lower confidence in the conclusions drawn from the results.  Participants with sub-optimal childhood experiences and idealisation or anger against parents can be influenced by this in their recall. |
| Cui et al., 2019, | Cross-sectional, cannot infer causation. It may that students who are struggling with anxiety and depression causes their parents to be more controlling and overprotective, thus acting in a helicopter parent manner. | Most of the students from the US sample were students in human and social sciences with the majority being female (89%). In the sample from Finland the majority of the participants were female (87.7%) and from social and educational sciences. Cannot be generalized to men or those in other fields of study. |  | Self-report.  Response bias.  Mono-method bias.  Depressed or anxious students may have altered perceptions of their parents.  It is possible that those students who most value autonomy perceive their parents as engaging in more hovering behaviours than other students  Measure asked whether at least one parent acted in a controlling way, but did not differentiate between mother and father. Parents can have major differences in their parenting style. |
| Darlow et al., 2017 | The data was only collected at one point in time and therefore no conclusion about helicopter parenting and adjustment to college over time can be drawn.  Correlational data, can be that struggling students causes overprotective parents. | Majority of the sample was female 241 out of 294. Cannot be generalized to the male population.  All the participants were psychology students from a medium-sized state university. Cannot be generalized to students within other disciplines or whom are attending different types of colleges and universities | Low range for  Depression (1.15-3.5) Self-efficacy (2.1-5.0)  Academic adjustment (1.48-7.74). Following correlations could be weakened by this reduced variability | All data consisted of self-report. Mono-method bias.  Depressed or anxious students may have altered perceptions of their parents.  It is possible that those students who most value autonomy perceive their parents as engaging in more hovering behaviours than other students.  Measure asked whether at least one parent acted in a controlling way, but did not differentiate between mother and father. Parents can have major differences in their parenting style.  The measure of student preference for parental intervention was developed for the present study. The reliability was somewhat low, thus tempering conclusions based on that measure. |
| Finkelstein et al., 2001 | Cross-sectional data, cannot infer causation. It is possible that the rates of depression the girls has is the cause of the maternal behaviour.  Did not assess firm control by fathers or male guardians.  Assessment took place at the clinic. Could have affected baseline anxiety level. | The sample only consisted of girls. Cannot be generalized to boys.  Help-seeking/clinically-referred sample. Cannot be generalized.  Groups were not equal in size African American = 50  Caucasian = 41  Hispanic = 20 | No relationship between firm control and depressive symptoms were found in the total sample. With ethnicity as a moderator high levels of control were predictive of fewer symptoms of depression among African Americans. However, can we trust this when considering the small sample size in each group? | All measures were self-report. Mono-method bias.  Have not reported the internal consistency of the measurements – thus, we cannot be confident in the conclusion drawn from the measurements.  36% (n=40) of the participants scored on the measure of depression that indicated a level of symptom endorsement associated with clinical depression. These participants could have a distorted perception of their parents due to clinical depression. |
| Finkenauer et al., 2005 | Correlational data, cannot infer causation. Adolescents problem behaviour can cause parental psychological control.  Questionnaires filled out in class, could affect how participants responded. | 88% of children lived with both parents – would the effect be the same in a single parent household? | The correlational design prevents from excluding the influence of third variables on the observed links between adaptive parenting and self-control, on the one hand, and emotional and behaviour problems and self-control, on the other. Consequently, the observed relationships could be invalid. | All measures were self-report from one single reporter. Mono-method bias.  Low internal consistency for the measurements: Strict control scale= .65  Psychological control scale= .68  Short version of self-control scale =.67  Behavioural problem= .68  Measurement of perceived parenting style does not distinguish between father and mother. Participants can have parents with very different parenting styles. We do not know if their response are related to one or both parent.  Participants with sub-optimal childhood experiences and idealisation or anger against parents can be influenced by this in their recall. |
| Gargurevich & Soenens, 2016 | Cross–sectional design, cannot make claims regarding direction of effects or causality.  Questionnaires filled out in class. Could have affected how they responded.  First author of study was present. Researcher expectancy could have affected the results | Peru is not representative of all of Latin America, can therefore not generalize the results beyond Peru |  | All measures were self-report. Response bias. Mono-method bias.  Internal consistency for depressive experiences was .69 (moderately low)  Participants with sub-optimal childhood experiences and idealisation or anger against parents can be influenced by this in their recall. |
| Goger, Rozenman & Gonazales, 2020 | Cross-sectional, cannot infer causation. Anxious students could cause their mothers to be controlling | The majority of the sample was female (76.6). Cannot be generalized to men.  Only asked participants to report on maternal parenting. Cannot be generalized to family dynamic as a whole. Fathers could have a different impact on the anxiety levels of the students. |  | Self-report. Response bias.  Mono-method bias. |
| Hong & Cui, 2019 | Cross-sectional study design cannot determine causation. | The majority of the participants were female (89.6%) and can therefore not be generalized to men. The participants were recruited from introductory courses offered by colleges of human sciences and social scienses. Cannot be generalized to students in other fields of study. |  | Self-report. Response bias.  Mono-method bias.  Depressed or anxious students may have altered perceptions of their parents. |
| Heider et al., 2008 | Cross-sectional study design cannot determine causation.  The comparability with studies using the original 25-item PBI scale is limited, because an abbreviated PBI-scale was used. Generally, shortening scales can reduce reliability | Groups were not equal in size  Belgium (n=2419)  France (n=2894)  Germany (n=3555)  Italy (n=4712)  Netherlands (n=2372)  Spain (n=5473) |  | Self-report. Response bias. Mono-method bias.  PBI retrospective recall of behaviour.  Participants with sub-optimal childhood experiences and idealisation or anger against parents can be influenced by this in their recall. |
| Inguglia et al., 2016 | Cross-sectional study design, cannot determine causation. | The US sample was predominantly female (72%). Gender bias. |  | Self-report. Response bias. Mono-method bias.  Participants were not allowed to report separate ratings for maternal and paternal parenting.  Participants with sub-optimal childhood experiences and idealisation or anger against parents can be influenced by this in their recall. |
| Klein et al., 2020 | Cross-sectional study design cannot determine causation. | The mean age was 59.8. Cannot be generalized to younger populations. | An ultra-short screening version of FEE was used. Short questionnaires can weaken the reliability. | Self-report. Response bias. Mono-method bias.  Retrospective report of parenting. Participants with sub-optimal childhood experiences and idealisation or anger against parents can be influenced by this in their recall.  Older participants are looking back on their parents parenting style-concerns. Later relationships with parents is bound to play a role in how they remember their childhood.  Have not reported the internal consistency of the measurements – thus, we cannot be confident in the conclusion drawn from the measurements. |
| Knappe et al., 2012 | Correlational associations, cannot infer causation. |  |  | Self-report. Response bias.  Assessment of parenting was retrospective recall.  Participants with sub-optimal childhood experiences and idealisation or anger against parents can be influenced by this in their recall.  Moderately low internal consistency for parental overprotection .69 |
| Kouros et al., 2017 | Cross-sectional study, therefore the direction of effects cannot be assessed. Psychological distress among emerging adults may elicit over-involvement and helicopter behaviours from their parents, which in turn may contribute to their child’s psychological distress. | All participants were emerging adults attending a private university and taking psychology course. Cannot be generalised to students who attend other universities and specialising in other disciplines.   Predominately female samples. |  | All measures were self-report. Response bias. Mono-method bias.   Did not differentiate between mothers’ or fathers’ parenting. Do not know if participants responded with one or both parents in mind.    Participants with sub-optimal childhood experiences and idealisation or anger against parents can be influenced by this in their recall.  Internal consistency of autonomy support was low = .62. Which can have weakened relations in the study. |
| Kullberg et al., 2021 | Cross-sectional, cannot infer causation. | The mean age was 49.7. Therefore the findings cannot be generalized to a younger population. |  | Self-report.  Response bias.  Mono-method bias.  Retrospective recall.  Participants with sub-optimal childhood experiences and idealisation or anger against parents can be influenced by this in their recall.  Older participants are looking back on their parents parenting style-concerns. Later relationships with parents is bound to play a role in how they remember their childhood. |
| Kullberg et al., 2020 | Cross-sectional, cannot infer causation. | The mean age was 50.84 and can therefore not be generalized to a younger population. |  | Self-report.  Response bias.  Mono-method bias.  Retrospective recall.  Participants with sub-optimal childhood experiences and idealisation or anger against parents can be influenced by this in their recall.  Older participants are looking back on their parents parenting style-concerns. Later relationships with parents is bound to play a role in how they remember their childhood. |
| LeMoyne & Buchanan, 2011 | Cross-sectional data, cannot infer causation. It could be that students with low levels of well-being, as well as high levels of anxiety/depression view their parents as more intrusive than other students. | Convenience sample of undergraduate students from one university |  | Self-report. Report bias. Mono-method bias.  Participants with sub-optimal childhood experiences and idealisation or anger against parents can be influenced by this in their recall. |
| Levitt et al., 2020 | Cross-sectional study design, cannot infer causation. It could be that adolescents that struggle with anxiety and/or depression cause parents to be more controlling.  Testing took place at school. Could have had an impact on baseline anxiety.  Researchers were present. Researcher expectancy bias. | Small sample size | Concern regards to the length of testing. Young adolescents could have become bored and not give accurate replies. Possibly measurement error. | Self-report.  Response bias.  Mono-method bias.  Were asked to give their response in regard to their mother or their father. This does not give us a full view of the family dynamic.  Participants with sub-optimal childhood experiences and idealisation or anger against parents can be influenced by this in their recall. |
| Lieb et al., 2000 | No direct interviews with fathers regarding social phobia and depression. Father was only interviewed if the mother was dead or not locatable. |  | Odds ratios let you see whether the presence of 1 variable leads to an increase in the chance of another variable also being present. But this, similarly to a correlation, means that we cannot imply causation. |  |
| Luebbe et al., 2018 | Cross-sectional data, cannot infer causation. | Sample recruited from one university known for its high academic standards. May not be generalized to students attending different universities.  Sample mainly consisted of students who lived away from home. HP behaviour could be experienced differently for those still living with their parents.  87% were European American |  | Self-report. Response bias. Mono-method bias.   Retrospective questionnaires.  Participants with sub-optimal childhood experiences and idealisation or anger against parents can be influenced by this in their recall. |
| Luis et al., 2008 | Cross-sectional study, cannot infer causation.  Experimental setting could have affected baseline anxiety.  Researcher expectancies could have affected the outcome. | Small sample size for each group. |  | Self-report. Response bias.  Moderately low internal consistency in the measurement of Anxiety (RCMAS) for the Mexico participants .69 |
| Mandara & Pikes, 2008 | Correlational study, cannot infer causation. It is possible that depressive symptoms cause adolescent girls to perceive their mothers as using more psychologically controlling practices.  Study was completed in class, could have affected the way participants responded. | Small sample size.  Predominately female participants.  All from lower SES background in one Midwestern city. Cannot be generalized. |  | All measurements were self-report. Response bias. Mono-method bias.  Participants with sub-optimal childhood experiences and idealisation or anger against parents can be influenced by this in their recall.  Low internal consistency for  ‘home organisation’ .62 |
| Moilanen & Lynn Manuel, 2019 |  |  |  | All measures were self-report. Response bias. Mono-method bias.  Did not distinguish from mother and father in questionnaire.  Participants with sub-optimal childhood experiences and idealisation or anger against parents can be influenced by this in their recall. |
| Overbeek et al., 2007 | Used DSM III so diagnostic criteria could have changed as the DSM V is the one currently used. |  | Included 11 mental disorders in their analysis, seem a bit like fishing in the data. Like they’re including everything in the hope that something is significant. Especially considering other studies have specialised due to specific parts of parenting effecting specific disorders.  Parenting behaviour only predicted the variances in mental health by very small amount (1-5%), does this even mean anything in the real world? This is interesting in terms of effect size as larger samples normally produce larger effects and yet for such a large sample the explained variances are tiny. If it had been conducted with a smaller sample would they have found a result at all? Could this have been related to just sampling until it was significant? |  |
| Reed et al., 2016 | Cross-sectional data, cannot infer causation. It could be that parents who perceive their emerging adult child to have poor self-efficacy take on extra responsibility in trying to resolve any arising issues. | Predominately female sample. Cannot be generalized to males. |  | Self-report. Response bias. Mono-method bias.  Only assessed maternal helicopter parenting behaviours. |
| Reilly & Semkovska, 2018 |  | Possible self-selection in who chose to take part in the study (69.2% previously depressed). Information sheet stated that mood would be a focus of the study.  Predominately female sample, cannot be generalized to male population. |  | Self-report. Response bias. Mono-method bias.  69.2% of the participants reported having previous experiences with depression. This could have distorted their perceptions of parenting behaviour. |
| Reitman & Asseff, 2010 | Cross-sectional study, cannot infer causation.  Parents were to complete the questionnaires independently, but this was not controlled for and they might have ‘compared notes’ which could rendered their responses more similar than would otherwise have been the case. | All ‘children’ were recruited form a psychology class. Cannot be generalized. |  | Retrospective  self-report. Response bias. Mono-method bias.  Low internal consistency for the PBI overprotection scale .62  Participants with sub-optimal childhood experiences and idealisation or anger against parents can be influenced by this in their recall. |
| Rogers et al., 2020 | Direction of effects could not be determined between psychological control and anxiety and/or depression |  |  | Self-report. Response bias. Mono-method bias. |
| Schiffrin et al., 2019 | Cross-sectional data, cannot infer causation. Could be that those emerging adults who exhibit less competence and autonomy as well as those who experience symptoms of depression and anxiety may elicit more HP behaviour. | Sample predominantly female and white upper class. Cannot be generalized. |  | Self-report. Response bias. Mono-method bias. |
| Schiffrin et al., 2014 | Cross-sectional data, cannot infer causation. | Focused on perception of helicopter parenting among mothers of female college student’s. The generalizability more applicable to mother-daughter dyads  Mainly recruited psychology students. |  | Self-report. Response bias. Mono-method bias.  Only measure maternal HP behaviour |
| Segrin et al., 2013 | Cross-sectional, cannot infer causation. | Predominantly female children and female parents who responded to the questionnaires.  Most parents were married. Cannot be generalized. | Models tested from the data cannot rule out the effects of unobserved third variables. | Self-report. Response bias. Mono-method bias.  The measurements the ‘child’ completed in regards to parenting did not specify if it was for their mother or their father.  Participants with sub-optimal childhood experiences and idealisation or anger against parents can be influenced by this in their recall. |
| Silove et al., 1991 | Cross-sectional, cannot infer causation. | Small sample size.   All participants were referred to a hospital out-patient anxiety management programme – not generalizable to the general public  Age range from 19-62 years in a small sample – not investigated age differences. | Overfitted table (table 1 in article). According to these results affectionless constraint in both parents lead to GA, however the model should have included at least 10 cases per variable, not 1-4 like in this table.  Cannot establish a timeline of exposure- is it long term exposure to these parenting styles which effects? Or only during a specific developmental stage? Is it something which if only happens when very young participants can ‘get over’? | Retrospective self-report. Response bias.  No issues with restriction in range as participants ages from 19-62. However, this does mean that older participants are looking back on their parents parenting style-concerns. Later relationships with parents is bound to play a role in how they remember their childhood. |
| Soenens et al., 2012 | Cross-sectional data. Cannot infer causation. Personality vulnerability to depression may elicit the use of psychological control by parents. Or increase perceptions of parents as being controlling.  University students collected the Belgian data from kids/parents at home whereas school teachers collected the South Korean data in school. Different types of people collected the data and in different environments. Many things could have affected 1 group and not the other. Difficult to compare the groups. |  | The 2 samples differed significantly in depression scores – not controlled for. Having a larger difference between the groups to begin with would create a larger effect size and make the significance seem more. | Retrospective self-report. Response bias. Mono-method bias.  Participants with sub-optimal childhood experiences and idealisation or anger against parents can be influenced by this in their recall. |
| Turner, Faulk & Garner 2020 | Cross-sectional study design cannot determine causation. Could be that university students who struggle with depression cause parents to act in an helicopter parenting manner. | All participants were psychology students. Cannot be generalized to those in a different field of study. The majority of participants were female (70%). Gender bias. |  | Self-report.  Response bias.  Mono-method bias.  Participants with sub-optimal childhood experiences and idealisation or anger against parents can be influenced by this in their recall. |
| Wenze et al., 2019 | Self-report measures were completed in the first author’s lab. This could have had an impact on how the participants responded.  Researcher was present. Researcher expectancy bias. | Small sample size.   All participants were recruited at an American liberal arts college. Cannot be generalized.  The majority of the participants were female (77.88%). Gender bias. | There were 5 missing data points, for which average score was added. | Self-report.  Response bias.  Mono-method bias.  Participants with sub-optimal childhood experiences and idealisation or anger against parents can be influenced by this in their recall. |
| Wu et al., 2018 | Cross-sectional study, cannot infer causation.  Used a translated questionnaire and while they mention the acceptable reliability and validity there’s still the worry as to if it’s culturally appropriate. In a Taiwanese culture is there more of a fear of shaming the family like there is in other cultures? So would they fear being laughed at more? After all the authors mention themselves that gelotophobia is effected by external conditions like culture/social structure.  Completed in groups. Could have affected how they responded. | Small age range, not generalizable to other age groups.  Purposive sampling. | Concern regards to the length of testing. As even only using the subscales from each questionnaire it gives them a lot of questions to answer (90) so the young adolescents might be bored and not give accurate replies. Possible measurement error. | Self-report. Response bias. Mono-method bias.  Only used sub-sections of their questionnaires so can results be applied to attachment/parenting styles/phobia as a whole?  Mentions that gelotophobia is effected by external conditions like extraversion but in no way try to consider the impact of this.  Does not distinguish between maternal and paternal parenting.  Participants with sub-optimal childhood experiences and idealisation or anger against parents can be influenced by this in their recall. |
